# Socioeconomic determinants of protective behaviors and contact patterns in the post-COVID-19 pandemic era: A cross-sectional study in Italy

**DOI:** 10.1371/journal.pcbi.1013262

**Published:** 2025-08-04

**Authors:** Michele Tizzani, Laetitia Gauvin

**Affiliations:** 1 ISI Foundation, Turin, Italy; 2 Department of Applied Mathematics and Computer Science, Technical University of Denmark, Copenhagen, Denmark; 3 IRD, UMR 215 Prodig, 5, course of Humanities, F-93 322, Aubervilliers Cedex, France; University of Warwick, UNITED KINGDOM OF GREAT BRITAIN AND NORTHERN IRELAND

## Abstract

Socioeconomic inequalities significantly influence infectious disease outcomes, as seen with COVID-19, but the pathways through which socioeconomic conditions affect transmission dynamics remain unclear. To address this, we conducted a survey representative of the Italian population, stratified by age, gender, geographical area, city size, employment status, and education level. The survey’s final aim was to estimate differences in contact and protective behaviors across various population strata, both of which are crucial for understanding transmission dynamics. Our initial insights based on the survey indicate that years after the pandemic began, the perceived impact of COVID-19 on professional, economic, social, and psychological dimensions vary across socioeconomic strata, extending beyond the epidemiological outcomes. This reinforces the need for approaches that systematically consider socioeconomic determinants. In this context, using generalized linear models, we identified associations between socioeconomic factors and vaccination status for both COVID-19 and influenza, as well as the influence of socioeconomic conditions on mask-wearing and social distancing. Importantly, we also observed differences in contact behaviors based on employment status while education level did not show a significant association. These findings highlight the complex interplay of socioeconomic and demographic factors in shaping protective behavior and contact patterns. Understanding these dynamics can contribute to the improvement of epidemic models and better guide public health efforts for at-risk groups.

## Author summary

COVID-19 outcomes have been shown to vary across different socioeconomic groups, but the mechanisms by which socioeconomic conditions influence transmission are not yet fully understood. To gain insight into these relationships, we conducted a contact survey representative of the Italian population, incorporating socioeconomic dimensions to capture potential differences in contact patterns and protective behaviors - two critical factors in the spread of COVID-19. This survey aimed to gather data on participants’ perceptions of the pandemic’s impact, and their adoption of non-pharmaceutical interventions (NPIs) such as mask-wearing and social distancing, vaccination status, and contact behavior patterns. By analyzing this data, we were able to identify associations between socioeconomic factors and vaccination status for both COVID-19 and influenza, as well as the impact of socioeconomic conditions on mask-wearing and social distancing practices. Notably, we observed variations in contact behaviors based on employment status, while education level did not show a significant association. In summary, the survey results enabled us to pinpoint specific socioeconomic conditions linked to variations in contact and protective behaviors. This understanding is crucial for identifying when and which socioeconomic factors should be considered in epidemic modeling to accurately capture the drivers, whether contact patterns or protective measures, of infectious disease transmission.

## Introduction

Socioeconomic status (SES) is a critical determinant of health outcomes, with far-reaching implications for both communicable and non-communicable diseases [1,2]. While a substantial research effort has been devoted to examining the relationship between socioeconomic disparities and the prevalence of non-communicable diseases [2–7], it is also evident that socioeconomic divides play an important role in the transmission of infectious diseases. The COVID-19 pandemic further emphasized the role of socioeconomic factors in determining health outcomes, reinforcing their importance even in the context of communicable diseases. Protective behaviors, both pharmaceutical and non-pharmaceutical, were seen to be mediated by socioeconomic factors. Non-pharmaceutical interventions (NPIs) such as social distancing were instrumental in reducing infection rates and alleviating the burden on the healthcare system [8,9]. However the extent to which they were adopted varied significantly across socioeconomic groups. Moreover, vaccination uptake was found to correlate with education level and household income in several countries [10]. These behavioral disparities may have contributed to varying epidemic outcomes across socioeconomic groups. In several countries, populations in lower SES areas experienced faster virus spread and transmission, putting them at greater risk of COVID-19 infection, hospitalization, and mortality compared to their higher SES counterparts [11,12].

In line with the studies previously mentioned, Italy’s experience with the COVID-19 pandemic has shown a heterogeneous impact across different socioeconomic strata right from the early stages [13,14]. During the lockdown and post-lockdown periods, COVID-19 case rates were higher in more deprived municipalities compared to less deprived ones. However, hospitalization and case fatality rates did not show significant differences based on deprivation levels during any of the studied periods [15]. In response to the pandemic, Italy implemented stringent measures and a regional tiered system of non-pharmaceutical interventions (NPIs) [16], which influenced population contact behaviors [17] and, consequently, the rate of COVID-19 transmission [18]. Even with uniform measures across the country, there was a heterogeneous behavioral response across socioeconomic groups, indicating that socioeconomic characteristics shaped direct and community-level responses to these interventions [19].

These observations during the pandemic stress the importance of analyzing behavioral disparities across socioeconomic groups to eventually ensure equitable pandemic preparedness and response. To better understand how specific socioeconomic status (SES) factors influence epidemic dynamics, it is essential to address the root cause - how SES shapes contact behavior, the primary driver of communicable diseases. The epidemiological significance of age-stratified contact patterns in evaluating the impact of human behavior during the COVID-19 pandemic has been extensively investigated [20,21]. Age has been demonstrated to encapsulate a significant proportion of the observed inter-direct variation in contact patterns, and empirical age-dependent contact matrices have been measured in various settings and used for epidemic modeling. Contact survey protocols have been implemented before the pandemic [22,23]. However, research on the effects of socioeconomic factors on contact behavior remains limited, even though socioeconomic factors have been shown to influence epidemic outcomes [24]. Post-pandemic studies in a few countries - such as the UK, the Netherlands, Switzerland, and Belgium - have examined average contact numbers stratified by demographic characteristics [25]. In parallel, some efforts have been made to develop more general contact matrices that incorporate variables beyond age [26]. However, the association between socioeconomic stratification and contact behavior in the post-pandemic period still remains understudied. In particular, the investigation of the association between contact patterns and SES in the Italian context is still missing. Moreover, a gap remains in understanding the associations between socioeconomic status (SES) and the adoption of protective behaviors after the state of emergency was lifted during the COVID-19 pandemic. Behaviors observed during the crisis were often heavily influenced by strict mandates and heightened perceived risk, making it challenging to fully disentangle underlying socioeconomic (SES) drivers from policy-driven compliance. Studying the post-pandemic period, characterized by reduced or absent restrictions and potentially altered public perception, allows for a clearer assessment of persistent SES-related behavioral patterns. Establishing this ‘new normal’ behavioral baseline across different SES strata is crucial for parameterizing epidemic models intended for future preparedness and for designing proactive, equitable public health strategies that account for enduring inequalities *before* the next infectious disease threat emerges. Therefore, investigating the interplay between SES, protective behaviors, and contact patterns years after the pandemic’s onset provides essential insights not just in the context of the COVID-19 pandemic, but for strengthening future pandemic readiness.

To this end, we conducted a contact survey representative of the Italian population, stratified by demographic and socioeconomic indicators, four years after the start of the COVID-19 pandemic. First, to have a broader picture of the heterogeneous impact of the COVID-19 pandemic on the well-being of participants across different socioeconomic strata, we examine their perceptions of the impact on their social, psychological, economic, and work status. Second, we assessed the associations of SES with contact and protective behaviors adopted by participants at the time of the study. This included their adherence to non-pharmaceutical preventive measures, and the contact behavior of participants according to the various demographic and socioeconomic stratification of the population. This enables us to show the existing links between SES and protective behaviors as well as contact patterns in the context of the post-pandemic period. It thus contributes to a better understanding of how SES-related differences may influence the spread of communicable diseases.

## Data and methods

### Data description

To assess and analyze disparities in protective and contact behaviors in the aftermath of the COVID-19 pandemic, we designed a survey targeting the Italian population. The survey was administered in March 2024 through the Computer Assisted Web Interviewing (CAWI) protocol [27], the answers to the contact survey are representative of a subset of the Italian population stratified by *sex*, *age*, *geographical area* (*northwest*, *northeast*, *center*, *southern*, and *islands* Italian regions), *education level* (university degree or not), *city size* (0–10^3^, 10^3^–10^6^, and more than 10^6^ inhabitants), and *employment status* (*employed* or *unemployed*). The sample of *N* = 1200 participants was provided by the survey agency DOXA S.p.A. and was designed to be representative of the Italian population. Stratification was based on annual ISTAT Census data (2023) for gender, age, and geographical region, as well as the 2021 ISTAT Multipurpose Survey for education level and occupational status. To validate the use of 2021 occupational data, DOXA compared it with the most recent available ISTAT statistics from 2022 and confirmed that the overall occupational distribution remained consistent. Survey weights were also supplied, but only for reference purposes, as they were designed to be close to 1, reflecting the representativeness of the sample based on the aforementioned criteria. Questions asked of the participants and descriptive statistics are reported in the supplementary material (S2 Text) and Table A in S1 Text respectively.

First, to evaluate the heterogeneity of the perceived impact of the pandemic, we asked participants whether they felt it had negatively affected their lives economically, professionally, socially, and mentally.

Regarding protective health behaviors, the survey assessed participants’ vaccination status for COVID-19 and influenza, asking whether they were vaccinated. This allowed us to gauge vaccination uptake across different population segments. Furthermore, we explored participants’ attitudes and adherence to NPI measures, such as mask-wearing and social distancing.

Lastly, the survey’s main objective was to collect participants’ contact behavior patterns. Participants were asked on a Tuesday to record the number of contacts they had during the latest week-end and week days, namely Sunday and Monday, whether contacts were direct (i.e. people met in person and with whom participants exchanged at least a few words or had physical contact) or indirect(e.g. people within a radius of 1.5 meters from the participants with whom the participant didn’t exchange any words or physical contact), and, in the case of direct contacts, also the age of the individuals. Additionally, they were asked to record the contact location from the following options *home*, *work*, *essential activities* (e.g., grocery shopping), *leisure activities* (e.g,. sports), *transport*, *health* (e.g., medical examination), *study*.

### Ethical statement

All participants in this study were recruited voluntarily and provided informed consent before participating in the online surveys. Informed consent was obtained prior to any respondent completing the first survey. All participants are informed about the rationale and topic of the survey. The right of the participant to refuse to participate without giving reasons was respected at all times. The survey was conducted online. The study was approved by the University of Turin Committee for Bioethics, Protocol number 0555653.

### Statistical model of participants’ behavior and perception

To analyze the association between individual-level factors and participants’ behavior, we leveraged generalized linear models described hereafter. The explanatory variables included both demographic and socioeconomic characteristics. On the demographic front, we considered factors such as *age*, *gender*, *city size*, and *geographic area*. These variables allowed us to capture the potential heterogeneity in experiences and behaviors across different population segments. We also incorporated socioeconomic determinants, including *employment status* and *education level*. These socioeconomic indicators served as proxies for the diverse social and economic conditions shaping individuals’ lives in the post-pandemic period.

All the explanatory variables were encoded as categorical, in particular, the age was stratified into five groups (18-24, 25-34, 35-44, 45-54, 55+), the gender was divided into male and female, the city size was layered by the number of inhabitants (0–10^3^, 10^3^–10^6^, and more than 10^6^ inhabitants), the geographic area (*geo_area*) was stratified into four, the *northwest*, *northeast*, *center*, *south* and *islands* Italian regions, employment was divided between *employed* and *unemployed* participants, and education was divided between participants with a university degree (*degree*) and participants without (*nodegree*).

We focused on three types of outcome variables: participants’ perceptions of the pandemic’s impact, the protective behaviors they adopted, and the number of contacts they had, distinguishing between direct and indirect contacts.

Prior to conducting the regression analyses, we assessed the associations among the categorical independent variables using Cramér’s V [28]. The resulting heatmap (Fig A in S1 Text) illustrates the strength of pairwise associations. Overall, associations between predictors included in the same model were low, with the highest values observed between age group and employment status (0.24) and between working status and city size on weekdays (0.25). These levels of association are sufficiently low to proceed without concern for multicollinearity.

The reference group (intercept) for all the models was defined by the following category: age [35–44], education level [No degree], city size [1,000–10,000], geographic area [South], and employment status [Unemployed].

#### Perception of the impact of COVID-19.

To assess the perception of the impact of COVID-19 on the participants, we employed a multivariate ordinal logistic regression. The participants were asked whether they agreed with the statement that COVID-19 had a negative impact on their well-being in the economic, work, psychological, and social conditions. In this case, the ordinal outcome *Y* of the model is the participants’ answer measured on the Likert scale, Y∈{disagree<neutral<agree}. Formally, considering P(Y≤k) the cumulative probability of the event Y≤k, with k=1,2, represents the level of disagreement with the statement, the ordinal linear regression model can be written as

$$logit(P(Y\leq \text{k}))=log\left(\frac{P(Y\leq \text{k})}{P(Y>\text{k})}\right)=\beta_{k,0}+{𝐗}^{T}\beta$$ (1)

where

$$\beta =[\begin{array}{c} \beta \text{age}\\ \beta \text{gender}\\ \beta \text{city\_size}\\ \beta \text{geo\_area}\\ \beta \text{employment}\\ \beta \text{education} \end{array}]$$ (2)

is the vector of coefficients, and

$$𝐗=[\begin{array}{c} x_{\text{age}}\\ x_{\text{gender}}\\ x_{\text{city\_size}}\\ x_{\text{geo\_area}}\\ x_{\text{employment}}\\ x_{\text{education}} \end{array}]$$ (3)

is the vector of explanatory variables. In what follows, we will use the same explanatory variables as in Eq 3 unless specified otherwise.

#### Protective behavior.

Participants’ behavior toward protective measures was evaluated through questions about the number of vaccination intakes for COVID-19 and influenza, mask-wearing attitude, and adherence to social distancing. Social distancing and mask-wearing attitudes were assessed with an ordinal logistic regression model. The ordinal outcome *Y* was measured on a Likert scale, Y∈{never<sometimes<always}. Formally, considering P(Y≤k) the cumulative probability of the event Y≤k, where k=1,2, the ordinal linear regression model can be written as

$$logit(P(Y\leq \text{k}))=log\left(\frac{P(Y\leq \text{k})}{P(Y>\text{k})}\right)=\beta_{k,0}+{𝐗}^{T}\beta$$ (4)

Vaccination uptake was modeled with a logistic regression where the number of vaccination doses was mapped into a binary variable *Y* (*yes* or *no*) (since the focus was more on the attitude towards vaccination rather than the specific number of doses) and the model is expressed by equation

$$logit(P(Y=\text{yes}))=log(\frac{P(Y=\text{yes})}{P(Y=\text{no})})=\beta_{yes,0}+{𝐗}^{T}\beta$$ (5)

were β and **X** are expressed by Eqs (2) and (3).

#### Model assumptions and robustness checks for protective behavior and perception model.

To verify the proportional odds assumption for the ordinal models (*perception of impact of COVID-19* Eq (1) and *protective behavior* Eq (4)), we implemented the Brant test [29]. The results of the Brant test indicated no evidence against the proportional odds assumption, suggesting that the ordinal regression model was reasonable for our data. Details on how each type of outcome variable was incorporated into the models are described below. The analysis was performed using the MASS package for R [30]. To assess the robustness of these associations, Bayesian models (ordinal for NPIs, logistic for vaccination) were also implemented. These yielded results consistent with the frequentist approach, as detailed in the supporting information section (Sect 2.2 in S1 Text). We retained the frequentist approach in the main text due to this being a more common way to present the results.

#### Contact behavior.

For contact behaviors, we directly opted for a Bayesian approach. Modeling contact counts can involve substantial overdispersion and smaller effective sample sizes within subgroups, making Bayesian methods preferable for providing more stable estimates and richer uncertainty quantification. The dependent variable in this case was the total number of contacts *Yi* for each participant *i* and the model can be formally expressed by

$$Y_{i}∼\text{NegBin}(\mu_{i},r)$$ (6)

and the link function

$$\log(\mu_{i})=\beta_{0}+{𝐗}^{T}\beta$$ (7)

where β and **X** are defined by Eqs 2 and 3, $\mu_{i}$ the mean number of contacts for each participant *i*, and *α* is the reciprocal dispersion parameter: the higher *α* is the closer *Y*_*i*_ is Poisson distributed. We considered weakly informative priors (Gaussian distributed) for the coefficients β. We conducted the analysis separately for weekdays (Monday) and weekends (Sunday). This allowed us to investigate potential differences in contact patterns based on working and non-working participants. To discern the working behavior from the employment status, we integrated information on whether the participant had worked on the respective day into the employment variable. This resulted in considering three categories instead of one in the explanatory variable about the employment status: *working-employed*, i.e., participants who were employed and worked on the day of interest (weekday or weekend), *non-working-employed*, i.e., employed participants who did not work on the day of interest, and *unemployed* participants. Furthermore, we built separate models for each specific contact location, such as essential and leisure activities, transport, or health. By selecting only the contacts occurring in a particular setting, we could explore the differences in the determinants of contact behavior within that specific location and understand whether heterogeneity in contact patterns varies across the different settings.

The analysis was performed with Bambi (BAyesian Model Building Interface) [31], a Python library for Bayesian modeling. To improve the reliability of the contact data analysis, we applied a two-step filtering procedure designed to exclude participants who reported implausibly high numbers of contacts. The procedure is detailed in the supporting information section (Sect 3 in S1 Text).

## Results

### Impact of COVID-19 on the perception of well-being

In this section, we focus on the perception of the impact of COVID-19 concerning four aspects of participants’ lives: professional, social life, psychological well-being, and economic well-being.

**Fig 1:**
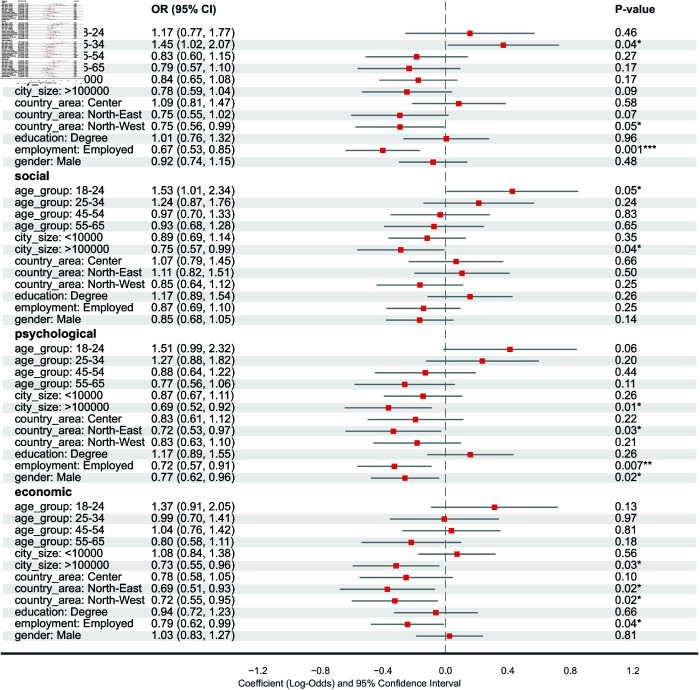
Ordinal logistic regression model for the perception of the impact of COVID-19 on work, social life, psychological well-being, and economic situation. P-value annotation legend: *: $1.00\times 10^{-2}<p<5.00\times 10^{-2}$, **: $1.00\times 10^{-3}<p<=5.00\times 10^{-2}$, ***: $1.00\times 10^{-4}<p<=1.00\times 10^{-3}$. The table shows the names of the explanatory variables (Variable), the odds ratio (OR), the confidence interval for the odds ratio (2.5% and 97.5%), and the estimated values of the coefficients of the model with their confidence interval (Est.)

Table 1 shows the total number and the percentage of the participants’ answers about their perception of the negative impact of the pandemic from an economic, psychological, social, or professional point of view. Overall, approximately 20% of participants reported being neutral over the perception of the negative impact of the pandemic in all domains. Around 40% of the participants recognized a negative influence on their economic, social, and psychological well-being, while around 30% of the participants disagreed with the statement. To further understand the mechanism behind the perception of the impact of COVID-19 on participant well-being, and the differences among socioeconomic and demographic groups, in Fig 1 we show the results of the four ordinal logistic regressions. Notice that the percentages in the description of the results are computed from the odds ratio derived from the statistical model.

**Table 1:** Descriptive statistics of the answers about the perception of the negative impact of COVID-19.

Impact Domain	Response	Count	Percentage (%)
Economic Impact	agree	479	39.92
	disagree	401	33.42
	neutral	320	26.67
Psychological Impact	agree	550	45.83
	disagree	395	32.92
	neutral	255	21.25
Social Impact	agree	538	44.83
	disagree	383	31.92
	neutral	279	23.25
Work Impact	agree	359	29.92
	disagree	591	49.25
	neutral	250	20.83

#### Impact on work.

The perception of the negative impact of the COVID-19 pandemic on work varied significantly by age, geographical location, and employment status (Fig 1). Compared to the reference age group (35-44), participants aged 25-34 were significantly more likely to agree with the negative impact statement (OR=1.45, 95% CI=1.02–1.07). Participants living in northwestern regions were less likely to agree compared to those in the Center region (OR=0.75, 95% CI=0.56–0.99). Compared to employed participants, unemployed individuals were significantly more likely to agree (OR=1.49, calculated from Employed OR=0.67, 95% CI=0.53–0.85). Education level and gender were not significantly associated with perceptions of work impact in this model. Older adults and those living in northern regions were more likely to disagree with the perception of the negative impact of the pandemic, while unemployed individuals appeared to have relatively more negative perceptions of the work-related effects of the COVID-19 crisis.

#### Impact on social life.

Perceptions of the impact of the pandemic on social life varied significantly only by city size (Fig 1). Compared to participants living in cities with a population between 10k–100k, those living in cities with over 100,000 inhabitants were less likely to agree that the pandemic had a negative impact on their social life (OR=0.75, 95% CI=0.57–0.99). Youngest participants (age 18-24), were more likely to perceive a negative impact compared to the reference group (OR=1.53 95% CI=1.01–2.34). Geographic area, education, employment status, and gender did not show significant associations. Overall, younger age groups were significantly more likely to agree with perceptions of the negative impact of the pandemic on social life, while other factors like geography and employment status did not have a significant effect.

#### Impact on psychological well-being.

Perceptions of the negative impact of the pandemic on psychological well-being varied significantly by city size, geographic area, employment status, and gender (Fig 1). Compared to the reference city size (10k–100k), those in cities with over 100,000 inhabitants were less likely to agree (OR=0.69, 95% CI=0.52–0.92). Participants in the Northeast region were less likely to agree compared to those in the South (OR=0.72, 95% CI=0.53–0.97). Compared to employed individuals, unemployed participants were more likely to agree (OR=1.39, calculated from Employed OR=0.72, 95% CI=0.57–.91). Male participants were less likely to agree compared to females (OR=0.77, 95% CI=0.62–0.96). Age was a weakly significant factor in this model, with younger participants (18-24) perceiving a higher burden from the COVID-19 pandemic (OR=1.51, 95% CI=0.99–2.32). Perceptions of the pandemic’s impact on psychological well-being were influenced by age, gender, city size, and employment status, with younger, female, and unemployed participants living in smaller cities being more affected.

#### Impact on economic well-being.

Perceptions of the impact of the pandemic on economic status varied significantly by city size, geographic area, and employment status (Fig 1). Compared to the reference city size (10k–100k), participants in cities with over 100,000 inhabitants were less likely to agree with a negative impact (OR=0.73, 95% CI=0.55–0.96). Participants in the Northeast (OR=0.69, 95% CI=0.51–0.93) and Northwest (OR=0.72, 95% CI=0.55–0.95) were less likely to agree compared to those in the South regions. Compared to employed participants, unemployed individuals were more likely to agree (OR=1.27, calculated from Employed OR=0.79, 95% CI=0.62–0.99). Age, education, and gender were not significantly associated with perceptions of economic impact. Perceptions of the pandemic’s economic impact differed by city size and employment status, with participants living in more densely populated areas feeling less affected by the pandemic. However, unemployed individuals perceived a stronger economic impact of the pandemic than employed participants.

#### Socioeconomic and demographic effects on the perception of the COVID-19 impact.

Demographic and socioeconomic factors largely influenced the perception of the negative impact of the COVID-19 pandemic. Age emerged as a significant factor, with younger participants reporting experiencing the pandemic more intensely. The perceived impact appeared to diminish with increasing age, suggesting that older individuals may have been better equipped to cope with the challenges posed by the crisis [32]. Employment status also played a significant role, with unemployed individuals consistently reporting a stronger negative impact on their work, psychological, and economic well-being. Geographical differences were also observed, with participants in the northern regions perceiving a less pronounced impact on their work compared to those in the southern regions. These results may reflect the intrinsic regional differences in the economic and social stratification that might have been exacerbated by the pandemic. Gender also emerged as a factor influencing the perception of the psychological burden, with female participants reporting a higher level of agreement on the pandemic’s detrimental impact on their well-being. This suggests that women may have experienced greater mental health challenges during the crisis, confirming previous results during pandemic times [33,34]. Furthermore, the perception of the economic burden was influenced by city size, with those residing in larger urban centers reporting a lesser impact of the pandemic. This may be attributed to the greater economic resilience and resources available in larger metropolitan areas [35]. Collectively, these findings highlight the significant socioeconomic disparities in how individuals perceive the impacts of the COVID-19 pandemic, adding to the already observed disparities in the epidemic outcomes. This shows the importance of understanding socioeconomic differences even before a potential pandemic. Specifically, it is essential to investigate variations in protective and contact behaviors across socioeconomic statuses (SES). Such an understanding can help elucidate whether the observed disparities in pandemic outcomes are attributable to differences in these behaviors by SES.

### Protective behaviour

We measured protective behaviors by asking participants about their adherence to non-pharmaceutical interventions, such as social distancing and face mask-wearing, as well as their vaccination uptake for both COVID-19 and influenza.

**Fig 2:**
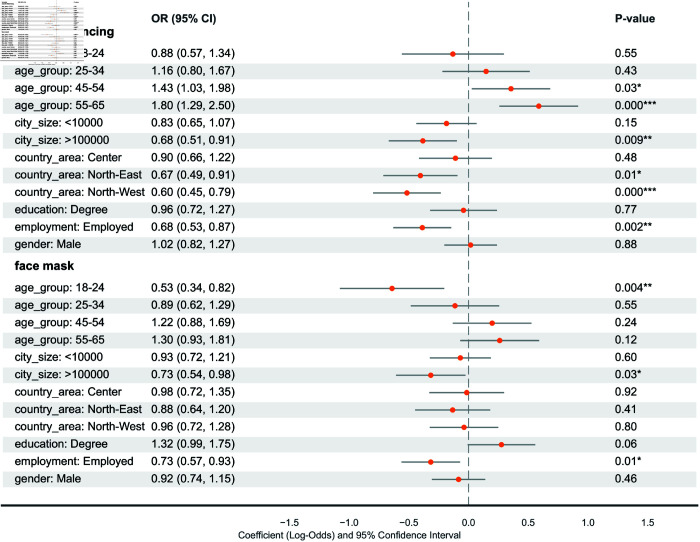
Ordinal logistic regression model for the non-pharmaceutical protective behavior. P-value annotation legend: *: $1.00\times 10^{-2}<p<5.00\times 10^{-2}$, **: $1.00\times 10^{-3}<p<=5.00\times 10^{-2}$, ***: $1.00\times 10^{-4}<p<=1.00\times 10^{-3}$. The table shows the names of the explanatory variables (Variable), the odds ratio (OR), the confidence interval for the odds ratio (2.5% and 97.5%), and the estimated values of the coefficients of the model with their confidence interval (Est.).

**Fig 3:**
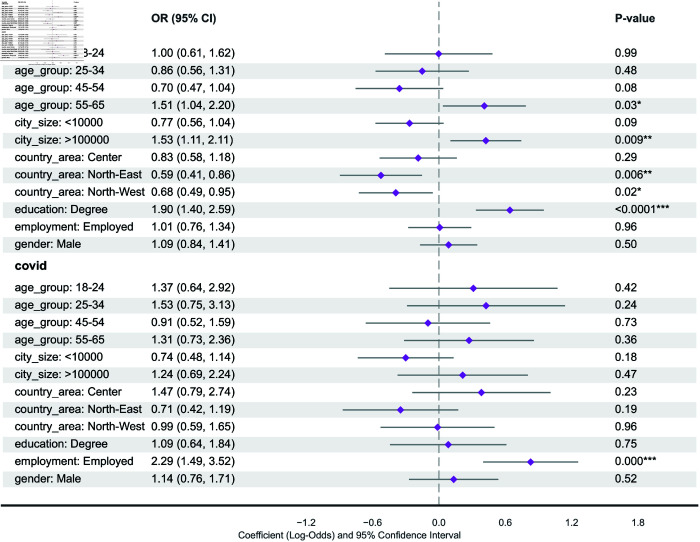
Binomial regression model for vaccination uptake. P-value annotation legend: *: $1.00\times 10^{-2}<p<5.00\times 10^{-2}$, **: $1.00\times 10^{-3}<p<=5.00\times 10^{-2}$, ***: $1.00\times 10^{-4}<p<=1.00\times 10^{-3}$. The table shows the names of the explanatory variables (Variable), the odds ratio (OR), the confidence interval for the odds ratio (2.5% and 97.5%), and the estimated values of the coefficients of the model with their confidence interval (Est.).

Table 2 presents the total number and percentage of participants who adopted various protective behaviors. Compatible with the official report from the Italian government [36], participants (over 90%) reported being vaccinated against COVID-19, likely due to the mandatory policies for healthcare workers and strongly encouraged policies for the rest of the population in place during the pandemic. Around 30% of the participants had received the influenza vaccine, 10% higher than the vaccine uptake for the general population reported by the Italian Minister of Health during the 2022-2023 flu season [37]. The adoption of non-pharmaceutical interventions (NPIs) like mask-wearing and social distancing was less consistent. The majority of participants either sporadically or never wore face masks, with about 10% reporting always using masks. Similarly, around 15% consistently practiced social distancing, while most did so occasionally or not at all. To further investigate the heterogeneity across demographic and socioeconomic strata, we implemented an ordinal logistic regression model for non-pharmaceutical intervention, shown in Fig 2, and a binomial regression model for vaccine uptake, Fig 3. Notice that the percentages in the description of the results are computed from the odds ratio derived from the statistical model.

**Table 2:** Descriptive statistics of the answers to the participants’ protective behavior.

Question	Response	Count	Percentage (%)
Covid vaccine	No	113	9.42
	Yes	1074	89.50
Flu vaccine	No	818	68.17
	Yes	366	30.50
Face mask	Always	119	9.92
	Never	510	42.50
	Sometimes	571	47.58
Social dist	Always	191	15.92
	Never	534	44.50
	Sometimes	475	39.58

#### Social distancing.

Older participants were more likely to always adhere to social distancing guidelines. In particular, those aged 45-54 (OR=1.43, 95% CI=1.03–.98) and 55-65 (OR=1.80, 95% CI=1.29–2.50) were significantly more likely to consistently comply [38], compared to younger participants (age group 35-44). Compared to employed individuals (reference), unemployed participants were more likely to comply (OR=1.47, calculated from Employed OR=0.68, 95% CI=0.53–0.87). Compared to the reference city size (10k–100k), those in cities >100k were less likely to comply (OR=0.68, 95% CI=0.51–0.91). Participants in the Northwest (OR=0.60, 95% CI=0.45–0.79) and Northeast (OR=0.67, 95% CI=0.49–0.91) were less likely to comply compared to those in the Southern regions.

#### Face mask.

Consistent face mask use was associated with employment status, education, and city size (Fig 2). Unemployed participants were more likely to consistently wear masks (OR=1.37, calculated from Employed OR=0.73, 95% CI=0.57–0.93). Participants with a university degree were more likely to wear masks compared to those without a degree (OR=1.32, 95% CI=0.99–1.75). Compared to the reference city size (10k–100k), those in cities >100k were less likely to consistently wear masks (OR=0.73, 95% CI=0.54–0.98). Younger participants (aged 18–24) were less likely to wear face masks than those in the 34-45 age group, controlling for all other factors.

#### Vaccine uptake.

COVID-19 vaccine uptake (Fig 3) varied significantly only by employment status. Unemployed individuals were significantly less likely to be vaccinated (OR=0.44, calculated from Employed OR=2.29, 95% CI=1.49–3.52). No other factors showed significant association in this model.

Influenza vaccine uptake (Fig 3) was associated with age, education, city size, and geographic area. Compared to the 35-44 age group (reference), participants aged 45-54 were less likely (OR=0.70, 95% CI=0.47–1.04, p=0.08) while those aged 55-65 were more likely (OR=1.51, 95% CI=1.04–2.20) to be vaccinated. Participants with a university degree were more likely to be vaccinated than those without a degree (OR for No Degree = 1/1.90 = 0.53, calculated from Degree OR=1.90, 95% CI=1.40–2.59). Those living in highly populated areas (>100k) were more likely to be vaccinated (OR=1.53, 95% CI=1.11–2.11). Participants in the Northeast (OR=0.59, 95% CI=0.41–0.86) and Northwest (OR=0.68, 95% CI=0.49–0.95) were less likely to be vaccinated compared to those in the Center region. It is important to note that the COVID-19 vaccine was mandatory for healthcare workers until November 2022, while the Green Pass, which restricted access to public enclosed spaces for unvaccinated individuals, was in effect for the rest of the Italian population until January 2023. We did not control for the occupation type of the participant, so the effect of being a healthcare worker cannot be captured by the analysis.

#### Socioeconomic and demographic factors of protective behavior.

Overall, participants’ ages were indicative of adherence to non-pharmaceutical interventions. Older participants were more likely to consistently adopt social distancing measures compared to younger participants. On the other hand, the analysis suggests that age did not play a strong role in the vaccine uptake, with only older participants (aged 55-65) more likely to be vaccinated against influenza compared to the reference group. Employment status also played a crucial role in adopting NPIs and vaccination. Unemployed participants were more likely to adopt NPIs consistently, such as face masks and social distancing, but less likely to be vaccinated against COVID-19. The geographic region also influenced vaccination rates. Participants from northeastern regions were less likely to consistently adopt social distancing compared to southern regions. Education level was another significant factor influencing vaccination decisions. Participants with a degree were more likely to be vaccinated against influenza. The results demonstrate that demographic, socioeconomic, and geographic characteristics show a strong association with how individuals adopt protective behaviors four years after the pandemic. This highlights the need to account for these factors when developing epidemiological models in order to more accurately capture disease transmission patterns and dynamics.

### Contact behavior and socioeconomic variables

Modeling the contact patterns of the population is crucial for understanding the transmission dynamics of communicable diseases. To this end, we conducted a two-fold analysis of the participants’ contact behavior. First, we provided an overview of the age-stratified contact matrices, which quantify the average number of contacts between individuals of different age groups. This descriptive analysis offered an overview of the overall structure and patterns of social interactions within the population several years after the beginning of the COVID-19 pandemic. Second, we employed negative binomial regression to investigate the factors potentially influencing contact behavior. With this approach, we explored how factors such as age, employment status, education level, and geographic location might shape social interactions.

#### Contact patterns.

**Fig 4:**
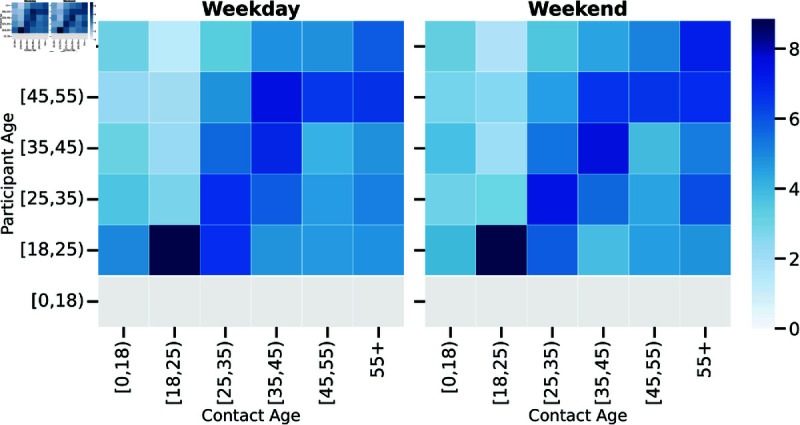
Contact matrices on weekday and weekend. The matrix element (*m*_*ij*_) is the median of 10000 bootstrap realizations of the average number of contacts of age group *i* with participants of age group *j*.

In this section, we describe the age-stratified contact patterns represented by the contact matrices. We consider the direct contact patterns of the participants on weekdays and weekends, first without differentiating with respect to the contact location, and subsequently, considering different contact locations. In Fig 4, we show the contact matrices for the two days of the surveys. Overall contact patterns show similar trends on both days, with more contacts on average during the weekend for younger participants. Younger participants have more interactions overall, in particular among the same age group, but also with the oldest age group. Considering different contact locations, Fig F in S1 Text and Fig G in S1 Text, overall, younger participants had more contacts than older ones for leisure activities and transportation, both on weekdays and weekends. Contacts on the weekdays during essential activities are mostly heterogeneous across the age groups. Contacts in health locations were mostly with older contacts for all the age groups, both on weekdays and weekends. Expectedly, homophilic contact patterns were more prevalent for leisure activities on both days, while contact patterns were more heterogeneous on public transport, especially on weekdays.

Furthermore, to provide context for post-pandemic social mixing, we examine the contact patterns from our study alongside those from the pre-pandemic POLYMOD [22] and pandemic-period COMIX [18] surveys in Italy. Fig D in S1 Text displays the age-stratified contact matrices from these three periods. A common feature observed across all three datasets is the pattern of age homophily, where individuals tend to have more contacts with people in their own or adjacent age groups. This tendency remains visible in our post-pandemic survey data. Additionally, examining our current study’s matrix (Fig D in S1 Text, right panel), we observe relatively heterogeneous mixing patterns, particularly involving older participant age groups (45-54 and 55-65), who report non-negligible contacts with various other age groups beyond their immediate diagonal neighbors. It is important to note that, differently from POLYMOD and COMIX, our survey does not cover participants older than 65 or younger than 18 years old, and the survey methodology was CAWI (Computer Assisted Web Interviewing) [27]. The methodological differences in the design of the three studies allow only a qualitative comparison of the contact patterns within each period. Further details on the methods employed can be found in the Supplementary Information (Sect 3.1 in S1 Text), with Fig D in S1 Text also reporting the explicit median and interquartile range values for each element.

#### Association of socioeconomic and demographic factors with contact patterns.

**Fig 5:**
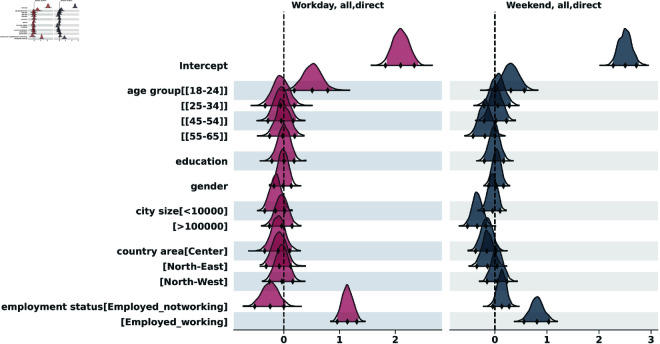
Highest Density Interval (HDI) of the posterior distribution for the coefficients (*β*) from the Bayesian negative binomial model for the number of direct contacts per participant on weekend and weekday. The y-axis shows the names of each category of the explanatory variables, excluding the reference categories used for the intercept: age_group[35-44], education[Nodegree], gender[Female], city_size[10000-100000], country_area[South], employment[Unemployed]. The x-axis represents the estimated coefficient value (log-scale effect) relative to the reference category. A 97,5% HDI excluding zero suggests a statistically significant difference from the reference group.

**Fig 6:**
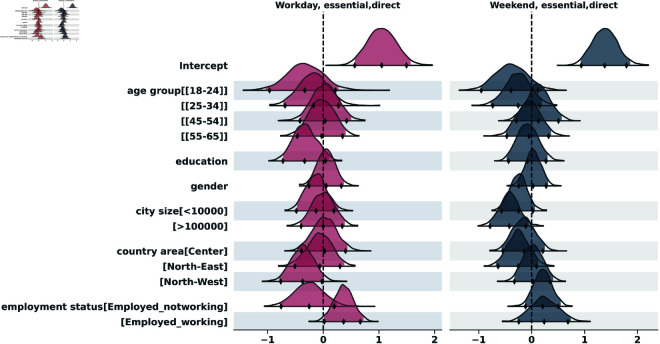
Highest Density Interval (HDI) of the posterior distribution for the coefficients (*β*) from the Bayesian negative binomial model for the number of direct contacts during essential activities, on weekend and weekday. The y-axis shows the names of each category of the explanatory variables, excluding the reference categories used for the intercept: age_group[35-44], education[Nodegree], gender[Female], city_size[10000-100000], country_area[South], employment[Unemployed]. The x-axis represents the estimated coefficient value (log-scale effect) relative to the reference category. A 97,5% HDI excluding zero suggests a statistically significant difference from the reference group.

**Fig 7:**
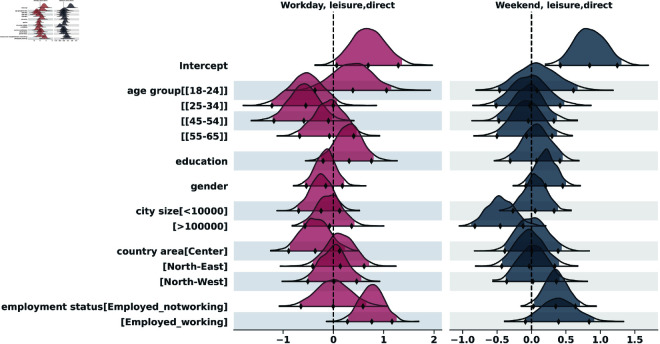
Highest Density Interval (HDI) of the posterior distribution for the coefficients (*β*) from the Bayesian negative binomial model for the number of direct contacts during leisure activities, on weekend and weekday. The y-axis shows the names of each category of the explanatory variables, excluding the reference categories used for the intercept: age_group[35-44], education[Nodegree], gender[Female], city_size[10000-100000], country_area[South], employment[Unemployed]. The x-axis represents the estimated coefficient value (log-scale effect) relative to the reference category. A 97,5% HDI excluding zero suggests a statistically significant difference from the reference group.

**Fig 8:**
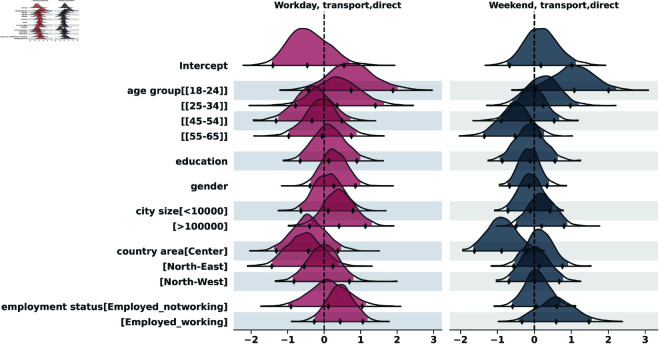
Highest Density Interval (HDI) of the posterior distribution for the coefficients (*β*) from the Bayesian negative binomial model for the number of direct contacts during transport, on weekend and weekday. The y-axis shows the names of each category of the explanatory variables, excluding the reference categories used for the intercept: age_group[35-44], education[Nodegree], gender[Female], city_size[10000-100000], country_area[South], employment[Unemployed]. The x-axis represents the estimated coefficient value (log-scale effect) relative to the reference category. A 97,5% HDI excluding zero suggests a statistically significant difference from the reference group.

**Fig 9:**
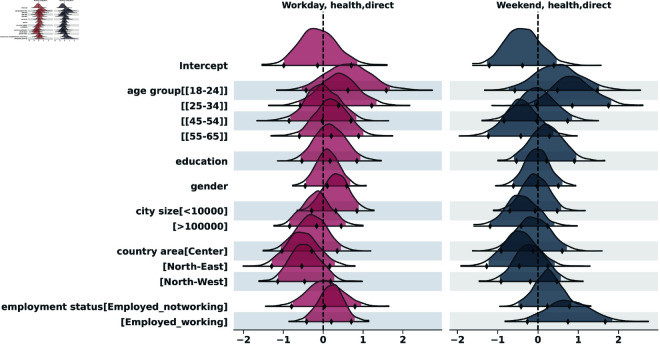
Highest Density Interval (HDI) of the posterior distribution for the coefficients (*β*) from the Bayesian negative binomial model for the number of direct contacts in health locations, on weekend and weekday. The y-axis shows the names of each category of the explanatory variables, excluding the reference categories used for the intercept: age_group[35-44], education[Nodegree], gender[Female], city_size[10000-100000], country_area[South], employment[Unemployed]. The x-axis represents the estimated coefficient value (log-scale effect) relative to the reference category. A 97,5% HDI excluding zero suggests a statistically significant difference from the reference group.

To analyze the distribution of contacts across the two survey days, we applied the negative binomial regression, both for indirect and for direct contacts. The results of the indirect contacts model are in the supporting information section (Sect 4.1 in S1 Text). Figs 5 to 9 show the posterior distributions of the model coefficients (*β*), which represent changes in the expected number of contacts on a logarithmic scale. This additive representation is particularly useful for visualization, as it enables clearer comparisons across categories.

In contrast, in the main text we report Incidence Rate Ratios (IRRs) [39], defined as exp(β), which describe the multiplicative change in the expected number of contacts relative to a reference category. An IRR with a 97,5% Highest Density Interval (HDI) that does not include 1 indicates a statistically significant difference from the reference group at the 2,5% level. IRRs are used in the resullt’s description because they more intuitively convey the magnitude of differences in contact numbers.

Results are reported for total contacts on weekdays (Table D in S1 Text) and weekends (Table F in S1 Text), as well as for contacts occurring in specific contexts: essential activities (Table H in S1 Text, Table J in S1 Text), leisure activities (Table L in S1 Text, Table N in S1 Text), transport (Table P in S1 Text, Table R in S1 Text), and health-related settings (Table T in S1 Text, Table V in S1 Text).

The reference group used in all models is: Age 35–44, Female, No degree, living in a city with 10,000–100,000 inhabitants, located in the South region, and currently unemployed.

#### Overall contacts.

Age showed a strong association with contact numbers (Table D in S1 Text, Table F in S1 Text). Compared to the reference group [35-44], participants aged [18-24] had significantly more contacts on weekdays (IRR = exp(0.51)≈1.67; 97,5% HDI = [1.23–2.39]) and weekends (IRR = exp(0.31)≈1.36; 97,5% HDI = [1.01–1.86]). This suggests about 67% more contacts on weekdays and 36% more on weekends for the youngest group. Older participants aged [55-65] had contact numbers similar to the reference group on weekdays, but potentially fewer contacts on weekends (IRR = exp(−0.19)≈0.83; 97,5% HDI = [0.66–1.03], not significant as HDI includes 1). Employment status was a key determinant. Compared to unemployed participants (reference), employed individuals who worked on the survey day had significantly more contacts on weekdays (IRR = exp(1.14)≈3.13; 97,5% HDI = [2.61–3.82]) and also on weekends (IRR = exp(0.81)≈2.25; 97,5% HDI = [1.73–2.89]). Employed individuals not working on the survey day had fewer contact with respect to the unemployed on weekdays (IRR = exp(−0.25)≈0.78; 97,5% HDI = [0.59–1.03]) and rather similar during weekends (IRR = exp(0.13)≈1.14; 97,5% HDI = [0.97–1.36]). Gender (Male vs Female reference) and Education (Degree vs Nodegree reference) did not show statistically significant associations with the overall number of contacts on either weekdays or weekends (Table D in S1 Text, Table F in S1 Text), as the 95% HDIs for their IRRs included 1.

Geographic differences were limited. Compared to the South (reference), only participants in the North-East showed a tendency towards fewer contacts, primarily on weekends (IRR = exp(−0.14)≈0.87; 97,5% HDI = [0.71–1.08], not significant) (Table F in S1 Text).

City size showed a significant association only on weekends for the largest cities. Compared to the reference size (10k-100k), those in cities >100k reported significantly fewer contacts (IRR = exp(−0.34)≈0.71; 97,5% HDI = [0.58–0.85]) (Table F in S1 Text).

#### Essential activities.

In Fig 6 we show the results of the negative binomial regression for contacts during essential activities, details are shown in the supporting information section (Sect 4.2 in S1 Text). Younger individuals ([18-24]) tended to have more contacts than the reference [35-44] group, though the effect was not statistically significant. Employed working participants had significantly more contacts than unemployed on weekdays (IRR ≈exp(0.36)=1.43; 97,5% HDI = [1.02–2.05]). Those in the largest cities (>100k) had significantly fewer contacts than the reference size on weekends (IRR = exp(−0.42)≈0.66; 97,5% HDI = [0.45–0.93]).

#### Leisure activities.

In Fig 7 we show the results for contacts that took place during leisure activities. Age effects were present, with the [18-24] group having more contacts than the [35-44] reference on weekdays, though not always statistically significant (Weekday IRR = exp(0.47)≈1.60; 97,5% HDI = [0.71–3.76]). On weekdays, employed participants who were working reported significantly more contacts than unemployed individuals (IRR = exp(0.74)≈2.10; 97,5% HDI = [1.27–3.46]). A similar trend was observed on weekends, where employed individuals - regardless of whether they were currently working - also had more contacts. Although the HDI for this effect does not lie entirely above zero, the majority of the mass supports a positive association. More detailed information is reported in the supporting information section (Sect 4.3 in S1 Text).

#### Transport.

Fig 8 shows the results for contacts on public transport. Age effects were very strong here. Compared to the [35-44] reference group, the [18-24] group had dramatically more contacts on weekdays (IRR = exp(2.06)≈7.85; 97,5% HDI = [2.27–34.8]) and weekends (IRR = exp(1.08)≈2.94; 97,5% HDI = [1.03–9.10]). Employed working individuals had significantly more contacts than the unemployed on weekdays (IRR = exp(1.33)≈3.78; 97,5% HDI = [1.43–10.47]). Residents in the North-West had significantly more transport contacts than Southerners on weekdays (IRR = exp(1.07)≈2.92; 97,5% HDI = [1.06–7.77]).

#### Health locations.

In Fig 9 we show the results for contacts in health locations. Contact rates were generally lower. The [18-24] age group tended to have more contacts than the [35-44] reference, but this was not statistically significant. Employment status differences were generally not significant. Living in the largest cities (>100k) was associated with significantly fewer contacts on weekdays compared to mid-sized cities (IRR = exp(−0.73)≈0.48; 97,5% HDI = [0.23–1.01], borderline).

#### Comparison with indirect contacts.

We performed the same analyses for indirect contacts (Table E in S1 Text, Table G in S1 Text, Table I in S1 Text, Table K in S1 Text, Table M in S1 Text, Table O in S1 Text, Table Q in S1 Text, Table S in S1 Text, Table U in S1 Text, Table W in S1 Text). Generally, the main predictors identified for direct contacts also influenced indirect contacts in the same direction. For instance, the strong positive association of young age ([18-24]) and working employment status with weekday contacts was observed for both direct and indirect types, often with comparable effect sizes (IRRs), particularly in leisure and transport settings. Similarly, the significant negative association of large city size (>100k) with overall weekend contacts and health contacts was consistent across both direct and indirect measures. However, some subtle differences emerged, particularly on weekends, where certain factors significantly associated with direct contacts (e.g., age [18-24] for overall weekend or transport contacts) showed weaker or non-significant associations for indirect contacts. Overall, while minor variations exist, there were no fundamental differences suggesting distinct socioeconomic or demographic drivers for direct versus indirect contacts; factors influencing one type tended to influence the other similarly. Detailed results for indirect contacts are available in the Supplementary Information.

#### Socioeconomic determinants of contact behavior.

Relative to a baseline of [35-44] year old, unemployed females, with no degree in mid-sized Southern cities, age and employment status were the most consistent predictors of contact patterns. Younger individuals ([18-24]) reported significantly higher contact numbers overall (around 1.4-1.7 times the baseline) and dramatically higher contacts in transport settings (up to 8 times the baseline on weekdays). Employment status displayed a strong temporal pattern: employed individuals working on the survey day had substantially more contacts than unemployed individuals (around 2-3 times more, overall and in leisure/transport settings), especially on weekdays. Gender and education level showed limited association with contact frequency relative to their baselines. Geographic location and city size had context-dependent effects, with large cities sometimes associated with fewer contacts (overall weekend, essential, health) but potentially more transport contacts, and regional differences emerging primarily on weekends or for specific activities like transport. These findings underscore that contact behavior is shaped by a complex interplay of demographics (age), socioeconomic position (employment), and the specific context (day type, activity/location).

## Discussion

In this study, we conducted a survey disaggregated by socioeconomic and demographic strata, offering initial insights into the relationships between these determinants and the behaviors relevant to the spread of infectious diseases in Italy following the COVID-19 pandemic. To have a broader overview of the heterogeneous impact of the COVID-19 pandemic, we first looked at the association between the demographic and socioeconomic characteristics of the participants and their perceptions of the impact on their social, economic, psychological, and occupational situation.

Age emerged as a key demographic determinant, with younger individuals reporting a more intense experience of the pandemic’s effects, supporting the finding in the literature [40]. Gender also played a crucial role, particularly concerning psychological well-being, as female participants indicated a higher perceived burden, confirming previous studies [40]. Furthermore, the perception of the economic burden was influenced by the participants’ city of residence, with those living in larger urban centers reporting a lesser perceived impact on their economic situation. This may be indicative of the differential access to economic resources and opportunities between urban and rural areas, as well as the varying severity of pandemic-related disruptions in different geographical areas [35,41].

In terms of socioeconomic factors, employment status was a persistent determinant affecting all indicators of well-being measured as perception of a negative impact on the participants’ situation. Compatible with previous literature [40,42,43], unemployed participants reported experiencing a greater negative impact of the pandemic from the psychological point of view, compared to their employed counterparts. Employment status was also systematically associated with a perceived negative impact on economic and occupational well-being, as previously observed during the pandemic [44]. Adding to the literature about the heterogeneous impact of the pandemic, our study reveals the heterogeneous perceived impact of the pandemic’s burden across different socioeconomic statuses (SES) several years after the start of the pandemic. This highlights the necessity of accounting for these factors when considering the spread of infectious diseases. To this aim, we analyzed the association between socioeconomic determinants and both protective and contact behaviors, key elements in the infectious disease transmission dynamics, while controlling for other known important demographic factors.

Age was seen as a key determinant of adherence to non-pharmaceutical interventions (NPIs) in the study. Older participants were more likely to consistently adopt face masks and practice social distancing compared to their younger counterparts. This aligns with existing evidence that risk perception and health-protective behaviors tend to increase with age [45–47]. It is important to note that the survey was done in March 2024, when both flu and COVID-19 were still circulating among the population. In terms of contact behavior, younger participants reported higher overall contact levels compared to older age groups. This aligns with previous research indicating that younger individuals tend to have a richer social network [22,48]. These findings are similar to earlier research on how age relates to protective and contact behaviors, although that research focused on the post-pandemic period.

Turning to less explored factors, we found socioeconomic characteristics to be key determinants of the behaviors relevant to the spread of diseases. In particular, employment status exhibited an inverse relationship when comparing NPIs and vaccine uptake. Unemployed participants were more likely to consistently wear masks and maintain social distancing, potentially reflecting their heightened concerns about infection risk. However, they were less likely to be vaccinated against the virus. This stresses the multifaceted nature of vaccine hesitancy, which can arise from various socioeconomic barriers as seen in previous literature [49,50].

The analysis of contact patterns revealed significant heterogeneity driven primarily by age and employment status when compared against the middle-aged, unemployed baseline. Age was a major determinant, with the youngest adults ([18-24]) reporting substantially more contacts than the [35-44] reference group, aligning with prior research indicating that younger individuals tend to have richer social networks [22,48]. This pattern thus appears to have persisted despite the disruptions of the COVID-19 pandemic. Employment status appeared to be the most influential socioeconomic factor affecting contact patterns -an association also observed during the pandemic in a different national context [24]. This suggests a persistent link between employment and contact behavior that extends beyond the specific circumstances of the COVID-19 pandemic. Moreover, we uncover an additional layer of complexity, revealing a non-trivial relationship between employment status, weekdays, and weekends in relation to contact patterns. Working-employed participants were more likely to have higher contact levels on weekdays compared to unemployed individuals, who, conversely, reported more contacts on weekends than their non-working employed counterparts.

This finding strongly suggests that work-related activities are a primary driver of weekday contacts for the employed population, and the absence of these activities might alter contact structures even on weekends. Education level and gender, relative to the Nodegree/Female baseline, were generally not significant predictors of different contact behavior, although previous studies have noted gender differences in social interactions [51]. Geographic location and city size had more variable effects depending on the day type and contact setting. Understanding these determinants, especially the dominant role of age and the temporally-dependent effect of employment, is crucial for parameterizing realistic epidemic models [23,26]. Pragmatically, employment status emerged as a persistent factor shaping contact behavior, mask-wearing, and social distancing; instead, education was found to be linked to the influenza vaccination uptake.

Overall, these findings have significant implications for understanding the drivers of social patterns that underpin disease transmission. In particular, this study highlights the enduring influence of socioeconomic and demographic factors on pandemic-related experiences and behaviors in Italy, years after the initial crisis. Moreover, SES, particularly through employment status and education level, shapes not only the perceived burden of the pandemic but also dictates protective actions and contact patterns. The observed inverse relationship between NPI adherence and COVID-19 vaccination among unemployed individuals highlights a complex dynamic that warrants careful attention in the design of public health interventions [49,50]. Furthermore, the association between contact patterns and employment status, along with its variation across days of the week, points to the importance of integrating socioeconomic structures and temporal dynamics into epidemic models to enhance their accuracy and predictive value [24].

*Limitations:* While the survey and its analysis are an important step to assess the interplay between socioeconomic factors and contact and protective behaviors, it faces limitations due to the age range considered by the panel, which did not include participants younger than 18 years old and older than 65. Both these age groups possess distinct epidemiological relevance and may exhibit different behavioral patterns, particularly in the underage population. The second limitation was the use of a computer-assisted interview, which might create a biased sample of the population toward higher socioeconomic classes. Despite these limitations, our findings reveal significant heterogeneity in the associations between socioeconomic factors, perceptions, and behaviors, and call attention to the necessity of including these factors when modeling human behavior for communicable disease.

## Supporting information

S1 Text Includes detailed methods, robustness checks, descriptive statistics (Table A in S1 Text), multicollinearity checks (Fig A in S1 Text), participant distribution table (Table B in S1 Text), summary statistics for post-stratification weights Table C in S1 Text, Bayesian model results for perception and protective behavior (Fig B in S1 Text, Fig C in S1 Text), detailed contact matrices (Fig D in S1 Text, Fig E in S1 Text, Fig F in S1 Text, Fig G in S1 Text), Bayesian model for all direct and indirect contacts (Fig H in S1 Text–Fig Q in S1 Text) and full regression tables for contact models (Table D in S1 Text–Table W in S1 Text). (PDF)

S2 Text The survey questionnaire. (PDF)

S1 Data Contact data. (CSV)

S2 Data For weekdays and weekends. (CSV)

S3 Data Perception and protective behavior data. (CSV)
