# Supplementary material for: Socioeconomic determinants of protective behaviors and contact patterns in the post-COVID-19 pandemic era: A cross-sectional study in Italy
Source: PLoS Comput Biol. 2025 Aug 4;21(8):e1013262. doi: 10.1371/journal.pcbi.1013262 (PMC12338834; doi:10.1371/journal.pcbi.1013262)
Supplement: S1 Text — (PDF) [file pcbi.1013262.s001.pdf]

Supplementary Information for Socioeconomic determinants of protective behaviors and contact patterns in the post-COVID-19 pandemic era: a cross-sectional study in Italy

Michele Tizzani and Laetitia Gauvin

1 Data

1.1 Survey descriptive statistics

This section provides a summary of the key demographic and socioeconomic characteristics of the final survey sample (N=1200 participants). Table A presents the distribution of participants across different categories for the variables used in the analyses, alongside corresponding national distribution percentages derived from ISTAT data for comparison purposes. This allows for an assessment of the representativeness of the weighted sample.

**Variable**	**Category**	**Count**	**Sample (%)**	**National (%)**
Age group	18–24	132	11.00	11.2
	25–34	205	17.08	17.1
	35–44	237	19.75	19.5
	45–54	306	25.50	25.5
	55–65	320	26.67	26.7
Gender	Woman	599	49.92	49.9
	Man	601	50.08	50.1
Geographical area	Center	237	19.75	19.8
	Northeast	234	19.50	19.5
	Northwest	318	26.50	26.7
	South and Islands	411	34.25	34.0
Municipality size	<10,000	377	31.42	30.4
	10,000 - 100,000	540	45.00	46.5
	>100,000	283	23.58	23.1
Employment status	Not working	454	37.80	40.1
	Working	740	61.70	59.9
	No answer	6	0.50	–
Education	With degree	252	21.00	20.4
	Without degree	948	79.00	79.6

Table A. Descriptive statistics of the survey sample (N=1200). Shows the number and percentage of participants for each category across key demographic and socioeconomic variables, compared with national population distributions (%).

2 Methods

2.1 Assessment of Association Among Categorical Predictors

Before conducting regression analyses, we assessed the pairwise association strength between the categorical independent variables used in the models using Cramér's V statistic. Cramér's V ranges from 0 (no association) to 1 (perfect association). The results are shown in Fig A.

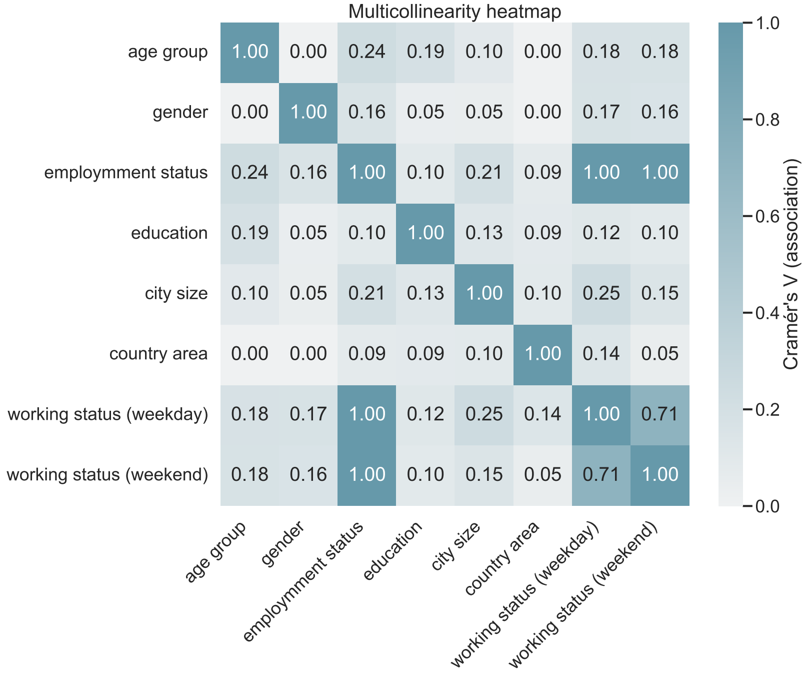

Fig A. Heatmaps of Cramér's V association statistic calculated for pairs of categorical predictor variables used in the regression models

2.2 Bayesian Robustness Checks for Protective Behaviors

To assess the robustness of the findings regarding protective behaviors (NPI adherence and vaccination uptake) presented in the main text (Figs 2 and 3 in the main text) using frequentist models, we conducted parallel Bayesian analyses. This approach allows for a probabilistic interpretation of the parameters and provides an alternative framework to verify the main conclusions derived from the frequentist approach.

Model Specification The Bayesian models were designed to mirror the structure of the frequentist generalized linear models described in the main text. For participant i, let Y_i be the outcome variable and X_i be the vector of predictor values. The predictor variables included were: 'age_group', 'education', 'gender', 'city_size', 'country_area', and 'employment'.

Ordinal Outcomes (NPI Adherence) For the ordinal outcomes measuring self-reported frequency of mask-wearing and social distancing ($Y_i \in \{1 = \text{never}, 2 = \text{sometimes}, 3 = \text{always}\}$), we specified a Bayesian cumulative logit model (proportional odds model). The probability of participant i reporting a response

in category k or lower ($P(Y_i \leq k)$) is modeled via the logit link function:

$$\text{logit}(P(Y_i \leq k)) = \alpha_k - X_i^T \beta \quad \text{for } k = 1, 2 \tag{1}$$

where α_k are the category-specific intercepts (cut-points) satisfying $\alpha_1 < \alpha_2$, and β is the vector of regression coefficients associated with the predictors X_i. The term $X_i^T \beta$ represents the linear predictor $\beta_{\text{age}} X_{i,\text{age}} + \beta_{\text{edu}} X_{i,\text{edu}} + \cdots + \beta_{\text{emp}} X_{i,\text{emp}}$. Note that the model estimates a single set of coefficients β across all cumulative probabilities (proportional odds assumption). This model was implemented using the 'cumulative' family with a 'logit' link in Bambi.

Binary Outcomes (Vaccination Uptake) For the binary outcomes representing vaccination uptake ($Y_i \in \{0 = \text{No}, 1 = \text{Yes}\}$ for COVID-19 and Influenza), we specified a Bayesian logistic regression model. The probability of participant i being vaccinated ($p_i = P(Y_i = 1)$) is modeled as:

$$Y_i \sim \text{Bernoulli}(p_i) \tag{2}$$

$$\text{logit}(p_i) = \beta_0 + X_i^T \beta \tag{3}$$

where β_0 is the intercept and β is the vector of regression coefficients for the predictors X_i. The term $X_i^T \beta$ again represents the linear predictor containing terms for each predictor variable. This model was implemented using the 'bernoulli' family with a 'logit' link in Bambi.

Reference Levels and Prior Distributions To ensure consistency in comparisons and interpretation, the reference levels for all categorical predictor variables were explicitly set within the data frame before model fitting using 'pandas.Categorical'. Following the specification used for the revised frequentist analysis presented in the main text, the reference levels were:

- `gender`: "Female"
- `education`: "Nodegree"
- `age_group`: "[35-44]"
- `city_size`: "10000-100000"
- `country_area`: "South"
- `employment`: "Unemployed"

We use the default, weakly informative priors provided by the Bambi library [1] for all model parameters (α_k, β_0, and components of β). These priors are generally chosen to be minimally influential on the posterior distribution while ensuring proper model behavior. Typically, they involve Normal distributions centered at zero with moderate variance for regression coefficients and Half-StudentT or Half-Normal distributions for any scale parameters (though less relevant here).

Implementation and MCMC Details The models were constructed and fitted using the Python library **Bambi** [1], which acts as a high-level interface to the probabilistic programming library **PyMC** for performing Markov Chain Monte Carlo (MCMC) sampling.

For each model, we generated posterior samples using the No-U-Turn Sampler (NUTS). We ran 4 independent MCMC chains, each with 1000 tuning steps followed by

2000 sampling draws, resulting in a total of 8000 posterior samples per parameter. Reproducibility was ensured by setting the random seed.

Convergence of the MCMC chains was assessed using the potential scale reduction factor, $\hat{R}$ (R-hat) [2], ensuring values were close to 1.0 (typically < 1.01) for all parameters.

Comparison with Frequentist Results The primary objective of these Bayesian analyses was to confirm the stability of the conclusions drawn from the frequentist models presented in the main text. We compared the inferences from the Bayesian models (based on posterior means and 95% Highest Density Intervals (HDIs) for the coefficients β) with the results from the frequentist models (based on point estimates/Odds Ratios and p-values/95% Confidence Intervals).

The Bayesian results were found to be highly consistent with the frequentist results reported in the main paper for both the ordinal NPI adherence models and the binary vaccination uptake models. Specifically, the directionality of the associations (i.e., whether a predictor was positively or negatively associated with the outcome) and the conclusions regarding statistical significance (i.e., whether a 97,5% HDI excluded zero, corresponding roughly to a frequentist p-value < 0.05) were concordant between the two approaches for all key findings.

This consistency across different statistical frameworks enhances our confidence in the robustness of the identified relationships between socioeconomic/demographic characteristics and protective health behaviors in the post-pandemic context described in the main text.

3 Contact data

To improve the reliability of our contact data analysis, we applied a two-step filtering procedure designed to exclude participants who reported implausibly high numbers of contacts.

Step 1: Filtering based on contacts at home by age group In the first step, we focused on the number of contacts participants reported having at home with individuals from various age groups. For each participant, we checked how many of these reported values were unusually high, specifically, above the 98th percentile for that type of contact. If a participant had more than one such extreme value, they were excluded. This helps remove responses that likely reflect overreporting or misinterpretation of the question, particularly regarding age-stratified contacts at home.

Step 2: Filtering based on overall contact levels across settings The second step considered all reported contacts, regardless of the setting (e.g, work, leisure...). For each setting-specific contact variable, we again identified values above the 98th percentile. We then counted, for each participant, how many times their responses exceeded these thresholds. Participants were excluded if they exceeded the threshold in more than three different contact contexts, indicating consistently extreme reporting.

Only participants who passed both filters—i.e., had a reasonable number of high contacts at home by age group and across all settings—were retained. This dual-filtering approach ensures the final dataset is not skewed by extreme or inconsistent responses and better reflects typical contact behavior. This results in a sample of 1001 participants.

3.1 Contact matrices

To further understand the contact behavior of the participants, and compare it with the pre-pandemic and pandemic period, we compute age-stratified contact matrices [3, 4].

Table B. Distribution of Participant Categories by Survey Day (N=1001 each day). Percentages are calculated based on the total number of participants after the filtering process.

**Variable**	**Category**	**Weekday Count(%)**	**Weekend Count(%)**
Gender	Female	515 (51.4%)	515 (51.4%)
	Male	486 (48.6%)	486 (48.6%)
Education	With degree	195 (19.5%)	195 (19.5%)
	Without degree	806 (80.5%)	806 (80.5%)
Age Group	[18-24]	92 (9.2%)	92 (9.2%)
	[25-34]	161 (16.1%)	161 (16.1%)
	[35-44]	194 (19.4%)	194 (19.4%)
	[45-54]	265 (26.5%)	265 (26.5%)
	[55-65]	289 (28.9%)	289 (28.9%)
Municipality Size	10000-100000	444 (44.4%)	444 (44.4%)
	<10000	325 (32.5%)	325 (32.5%)
	>100000	232 (23.2%)	232 (23.2%)
Geographical area	Center	192 (19.2%)	192 (19.2%)
	Northeast	206 (20.6%)	206 (20.6%)
	Northwest	260 (26.0%)	260 (26.0%)
	South and Islands	343 (34.3%)	343 (34.3%)
Employment status	Employed not working	147 (14.7%)	494 (49.4%)
	Employed working	456 (45.6%)	109 (10.9%)
	Unemployed	398 (39.8%)	398 (39.8%)

Participants recorded age information only for direct contacts, for this reason, we focus our analysis only on this subset. Contact matrix elements were computed according to the following equation:

$$m_{ij} = \frac{\sum_{t=1}^{T_i} w_{it} y_{ijt}}{\sum_{t=1}^{T_i} w_{it}} \tag{4}$$

where y_{ijt} denotes the reported number of contacts of the participant t of age i with someone of age j. T_i denotes all participants of age i, and w_{it} is the post-stratification weight for participant t.

These weights account for potential under- and over-sampling of specific demographic groups or survey characteristics relative to the target population. The summary statistics of the post-stratification weights for our study are shown in Table C. As noted earlier, these weights are intentionally close to 1 by design. To estimate average weekly contact patterns, we calculated a weighted average by assigning a weight of 5/7 to weekdays and 2/7 to weekends.

Table C. Summary statistics for post-stratification weights

**Statistic**	**Value**
Mean	1.00
Std. Deviation	0.06
Minimum	0.86
25th Percentile (Q1)	0.96
Median (50th Percentile)	1.00
75th Percentile (Q3)	1.04
Maximum	1.19

To account for sampling variability and obtain robust estimates for the matrix elements, we employed a non-parametric bootstrap procedure. Let $\mathcal{P}$ be the original sample of N participants. We generated $B = 10,000$ bootstrap samples, denoted as $\mathcal{P}_1^*, \mathcal{P}_2^*, \ldots, \mathcal{P}_B^*$. Each bootstrap sample $\mathcal{P}_b^*$ was constructed by drawing N participants from the original sample $\mathcal{P}$ with replacement.

For each bootstrap sample $\mathcal{P}_b^*$, we recalculated the contact matrix elements, denoted as $m_{ij}^{*(b)}$ (for overall contacts), using Equation (4), applied only to the participants included in $\mathcal{P}_b^*$. This resulted in a bootstrap distribution of B estimates for each element (i, j) of the matrix.

The final point estimate reported for each matrix element in the figures (Figures Fig D, Fig E, Fig G, Fig F) is the median of this empirical bootstrap distribution:

$$\hat{m}_{ij} = \text{median}\{m_{ij}^{*(1)}, m_{ij}^{*(2)}, \ldots, m_{ij}^{*(B)}\} \tag{5}$$

Furthermore, this procedure allowed us to estimate the statistical uncertainty; the interquartile range (IQR) of the bootstrap distribution $\{m_{ij}^{*(b)}\}_{b=1}^{B}$ is reported in parentheses in the detailed contact matrices presented (Figures Fig D-Fig G).

Contact patterns before, during, and after the COVID-19 pandemic. We present contact matrices for three distinct periods: pre-pandemic (POLYMOD), pandemic (COMIX), and post-pandemic (our current study). Given the differences in study design and methodology, we limit our analysis to a qualitative comparison of contact patterns. Across all three periods, age-based homophily in contact patterns is evident. However, the pandemic and post-pandemic matrices show greater interaction across age groups, as indicated by more populated off-diagonal elements. This effect is

especially pronounced among younger participants, who report more cross-age contacts in the post-pandemic period. It is important to note that, unlike POLYMOD and COMIX, our survey does not include participants under 18 or over 65 years old, and the data were collected through a computer-assisted web interview (CAWI) methodology.

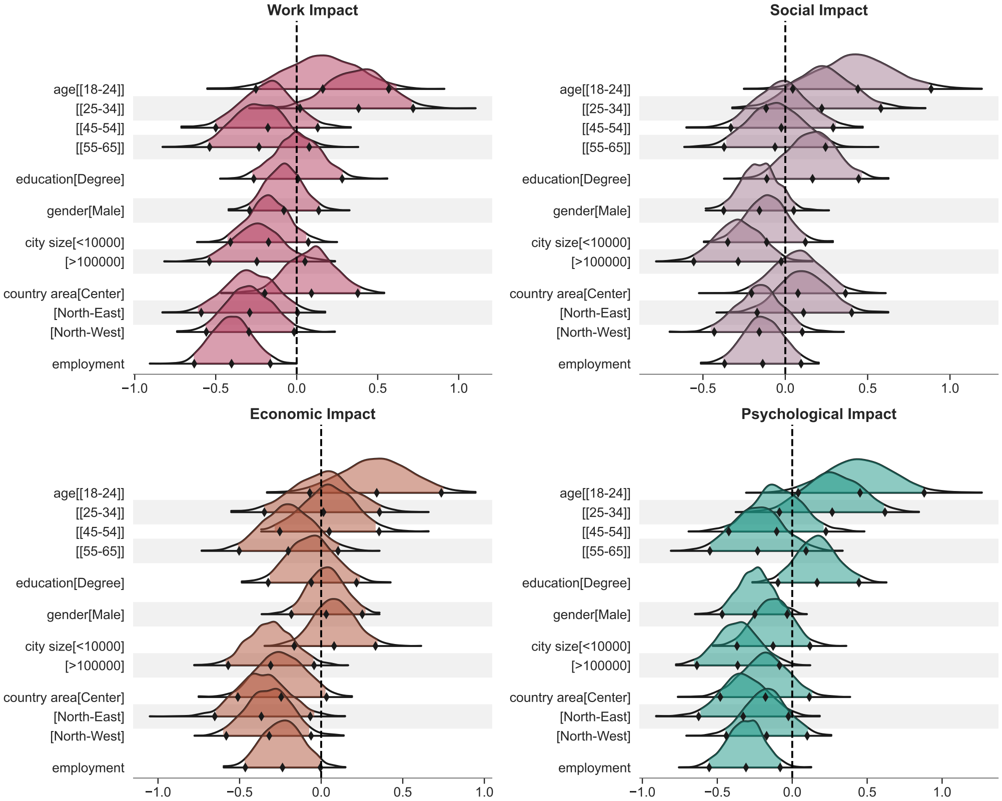

Fig B. Posterior distributions for the coefficients (β) from the Bayesian ordinal models assessing the perceived impact of COVID-19, corresponding to the frequentist models shown in Fig 1 (main text). Panels show results for perceived impact on Work (top left), Social Life (top right), Economic Situation (bottom left), and Psychological Well-being (bottom right). The y-axis shows the predictor categories compared against the reference group (Age [35-44], Gender Female, Education Nodegree, City Size 10k-100k, Country Area South, Employment Unemployed). The x-axis represents the estimated coefficient value (log-cumulative-odds) relative to the reference category (dashed line at zero), where higher values indicate stronger agreement with a *negative* impact compared to the reference. Distributions represent the posterior probability density, with points indicating the median and thick/thin lines showing the 50% and 95% Highest Density Intervals (HDIs), respectively. HDIs excluding zero suggest a statistically significant difference from the reference group.

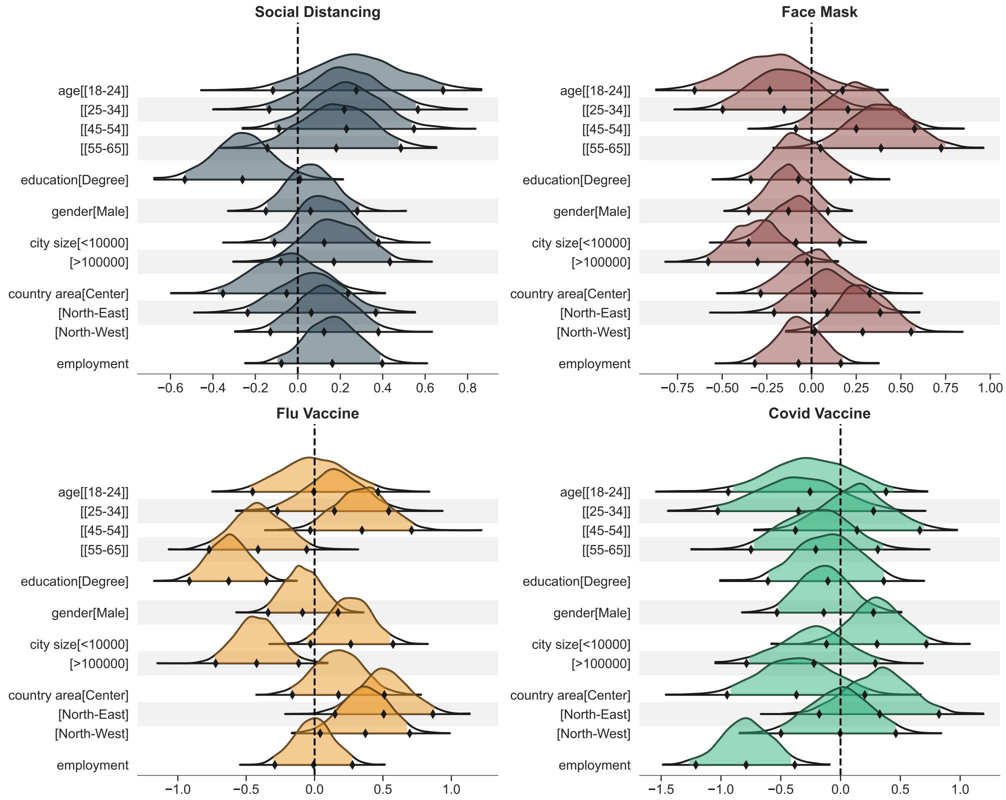

Fig C. Posterior distributions for the coefficients (β) from the Bayesian robustness check models for protective behaviors, corresponding to the frequentist models shown in Figs 2 and 3 (main text). **Top panels (Ordinal Outcomes):** Bayesian cumulative logit models for Social Distancing (left) and Face Mask use (right). **Bottom panels (Binary Outcomes):** Bayesian logistic models for Flu Vaccine uptake (left) and Covid Vaccine uptake (right). The y-axis shows the predictor categories compared against the reference group (Age [35-44], Gender Female, Education Nodegree, City Size 10k-100k, Country Area South, Employment Unemployed). The x-axis represents the estimated coefficient value (log-cumulative-odds for top panels, log-odds for bottom panels) relative to the reference category (dashed line at zero). Distributions represent the posterior probability density, with points indicating the median and thick/thin lines showing the 50% and 95% Highest Density Intervals (HDIs), respectively. HDIs excluding zero suggest a statistically significant difference from the reference group.

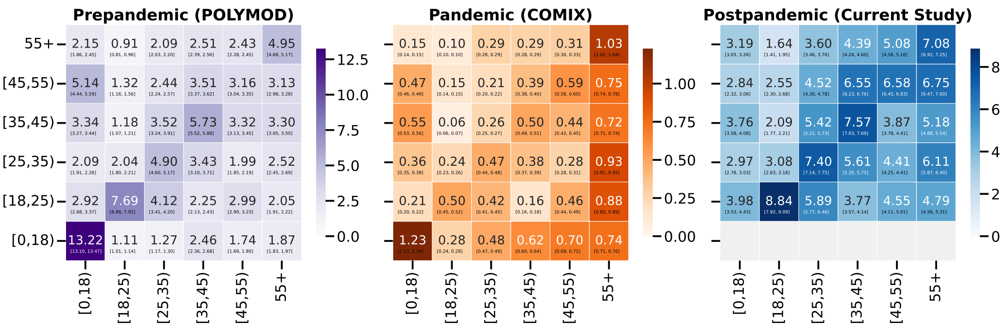

Fig D. Contact patterns before COVID-19 (Prepandemic), during COVID-19 (Pandemic), and after COVID-19 (Post-pandemic). The matrix element (m_{ij}) is the median of the bootstrap realizations of the average number of contacts of the age group i with participants of age group j. For each matrix, we consider only direct contacts over the whole period of the study. The interquartile range is also reported in the parentheses.

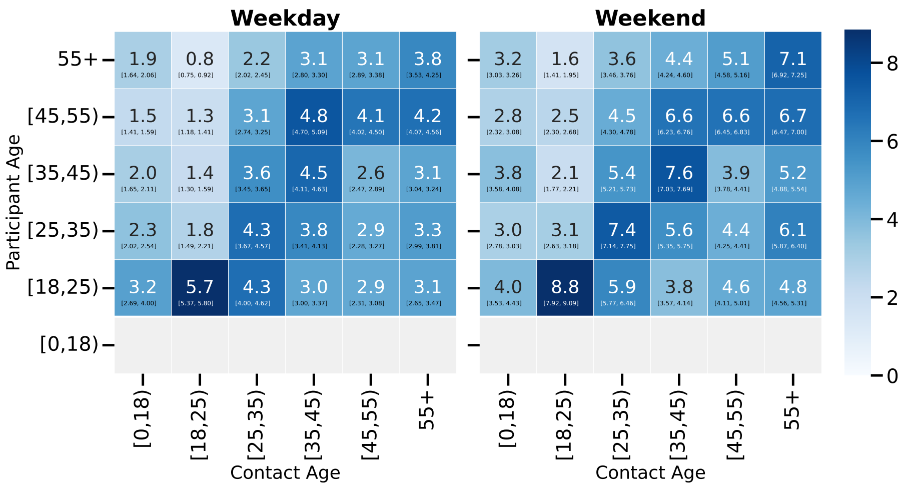

Fig E. Contact matrices on weekdays and weekends. The matrix element (m_{ij}) is the median of the bootstrap realizations of the average number of contacts of age group i with participants of age group j. The interquartile range is also reported in the parentheses.

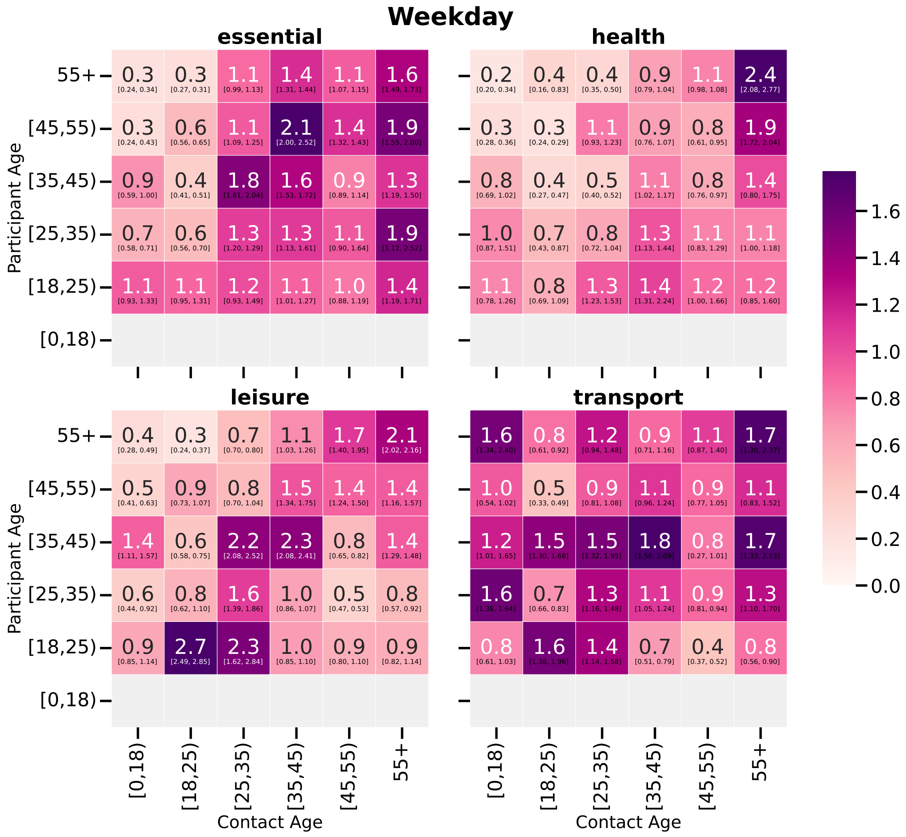

Fig F. Contact matrices for different contact locations for weekdays. The matrix element (m_{ij}) is the median of the bootstrap realizations of the average number of contacts of age group i with participants of age group j. The interquartile range is also reported in parentheses.

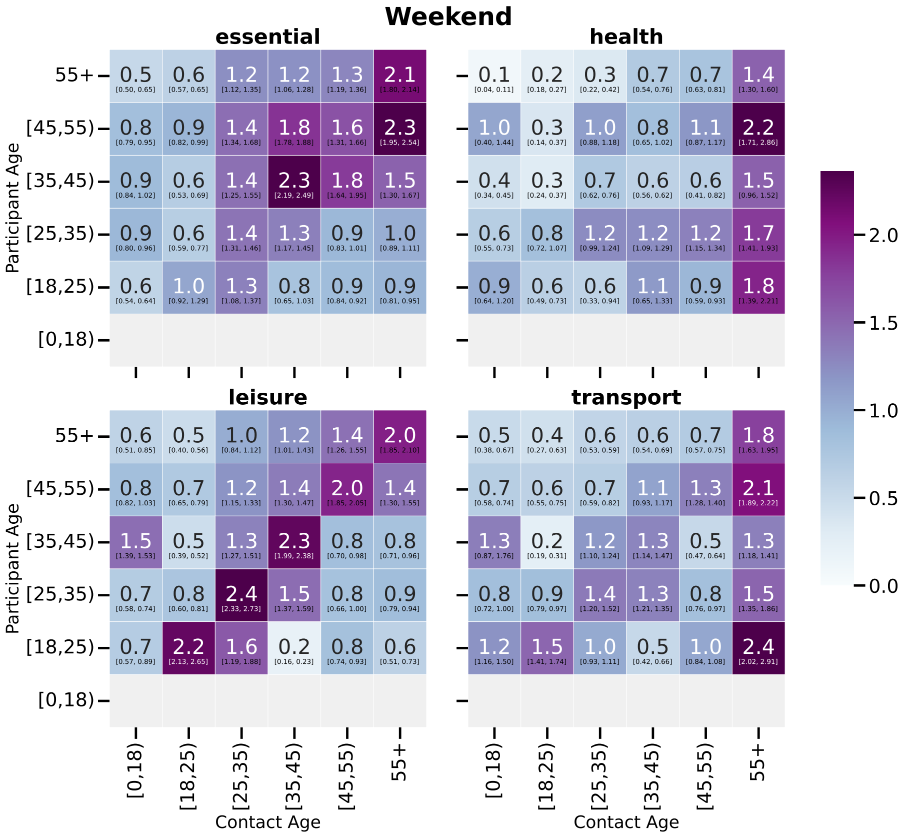

Fig G. Contact matrices for different contact locations for weekends. The matrix element (m_{ij}) is the median of the bootstrap realizations of the average number of contacts of age group i with participants of age group j. The interquartile range is also reported in the parentheses.

4 Results of the Bayesian negative binomial model for direct and indirect contacts

To estimate the Bayesian negative binomial regression model, we used Markov Chain Monte Carlo (MCMC) sampling techniques [5]. We specified non-informative prior distributions for the model parameters and ran multiple MCMC chains to ensure convergence. The MCMC samples were used to obtain posterior estimates of the model parameters and to quantify the uncertainty in these estimates. These diagnostic tests and model comparison methods ensured that our models met the necessary assumptions and provided a good fit to our data, allowing us to draw reliable conclusions from the results using Bayesian inference.

4.1 General settings

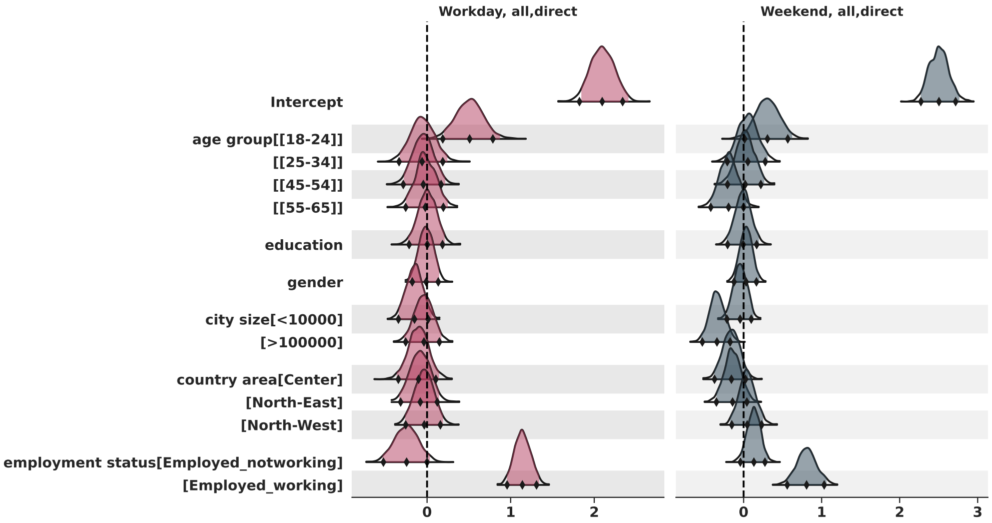

Fig H. Highest Density Interval (HDI) of the posterior distribution for the coefficients (β) from the Bayesian negative binomial model for the number of **direct** contacts per participant on weekend and weekday. The y-axis shows the names of each category of the explanatory variables, excluding the reference categories used for the intercept: age_group[35-44], education[Nodegree], gender[Female], city_size[10000-100000], country_area[South], employment[Unemployed]. The x-axis represents the estimated coefficient value (log-scale effect) relative to the reference category. A 97,5% HDI excluding zero suggests a statistically significant difference from the reference group.

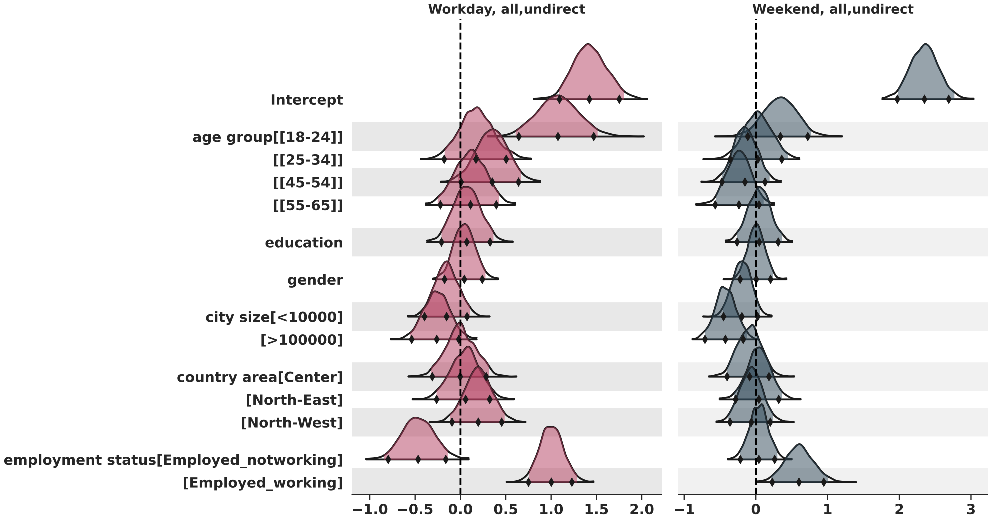

Fig I. Highest Density Interval (HDI) of the posterior distribution for the coefficients (β) from the Bayesian negative binomial model for the number of **indirect** contacts per participant on weekend and weekday. (Details as in Fig H)

	Mean	HDI 2.5%	HDI 97.5%
alpha	0.61	0.55	0.67
Intercept	2.10	1.85	2.40
age_group[[18-24]]	0.51	0.21	0.87
age_group[[25-34]]	-0.06	-0.32	0.24
age_group[[45-54]]	-0.05	-0.27	0.21
age_group[[55-65]]	-0.02	-0.25	0.24
education[Degree]	0.00	-0.22	0.20
gender[Male]	-0.01	-0.19	0.14
city_size[<10000]	-0.15	-0.34	0.05
city_size[>100000]	-0.04	-0.27	0.17
country_area[Center]	-0.10	-0.36	0.14
country_area[North-East]	-0.08	-0.33	0.14
country_area[North-West]	-0.04	-0.26	0.18
employed_working[Employed_notworking]	-0.25	-0.53	0.03
employed_working[employed]	1.14	0.96	1.34

Table D. Results of the Negative Binomial Model for direct contacts on Monday without a specific location

Weekday direct contacts

	Mean	HDI 2.5%	HDI 97.5%
alpha	0.34	0.30	0.37
Intercept	1.43	1.09	1.80
age_group[[18-24]]	1.08	0.61	1.51
age_group[[25-34]]	0.17	-0.17	0.57
age_group[[45-54]]	0.35	-0.02	0.66
age_group[[55-65]]	0.11	-0.25	0.42
education[Degree]	0.07	-0.20	0.36
gender[Male]	0.04	-0.18	0.27
city_size[<10000]	-0.15	-0.40	0.10
city_size[>100000]	-0.26	-0.56	0.02
country_area[Center]	0.00	-0.32	0.31
country_area[North-East]	0.05	-0.26	0.37
country_area[North-West]	0.20	-0.11	0.48
employed_working[Employed_notworking]	-0.46	-0.84	-0.14
employed_working[employed]	1.00	0.76	1.28

Table E. Results of the Negative Binomial Model for indirect contacts on Monday without a specific location

Weekday indirect contacts

	Mean	HDI 2.5%	HDI 97.5%
alpha	0.74	0.68	0.81
Intercept	2.50	2.28	2.76
age_group[[18-24]]	0.31	0.01	0.62
age_group[[25-34]]	0.05	-0.23	0.30
age_group[[45-54]]	0.02	-0.20	0.26
age_group[[55-65]]	-0.19	-0.42	0.03
education[Degree]	-0.01	-0.21	0.19
gender[Male]	0.03	-0.11	0.20
city_size[<10000]	-0.04	-0.21	0.12
city_size[>100000]	-0.34	-0.54	-0.16
country_area[Center]	-0.16	-0.37	0.05
country_area[North-East]	-0.14	-0.34	0.08
country_area[North-West]	0.05	-0.17	0.24
employed_working[Employed_notworking]	0.13	-0.03	0.31
employed_working[employed]	0.81	0.55	1.06

Table F. Results of the Negative Binomial Model for direct contacts on weekends without a specific location

Weekend direct contacts

	Mean	HDI 2.5%	HDI 97.5%
alpha	0.33	0.30	0.36
Intercept	2.35	1.99	2.76
age_group[[18-24]]	0.34	-0.15	0.76
age_group[[25-34]]	0.03	-0.34	0.44
age_group[[45-54]]	-0.15	-0.47	0.18
age_group[[55-65]]	-0.24	-0.55	0.10
education[Degree]	0.05	-0.26	0.36
gender[Male]	0.01	-0.22	0.23
city_size[<10000]	-0.20	-0.46	0.05
city_size[>100000]	-0.42	-0.70	-0.13
country_area[Center]	-0.09	-0.39	0.24
country_area[North-East]	0.04	-0.30	0.36
country_area[North-West]	-0.06	-0.38	0.23
employed_working[Employed_notworking]	0.04	-0.22	0.30
employed_working[employed]	0.60	0.22	1.00

Table G. Results of the Negative Binomial Model for indirect contacts on weekends without a specific location

Weekend indirect contacts

4.2 Essential activities

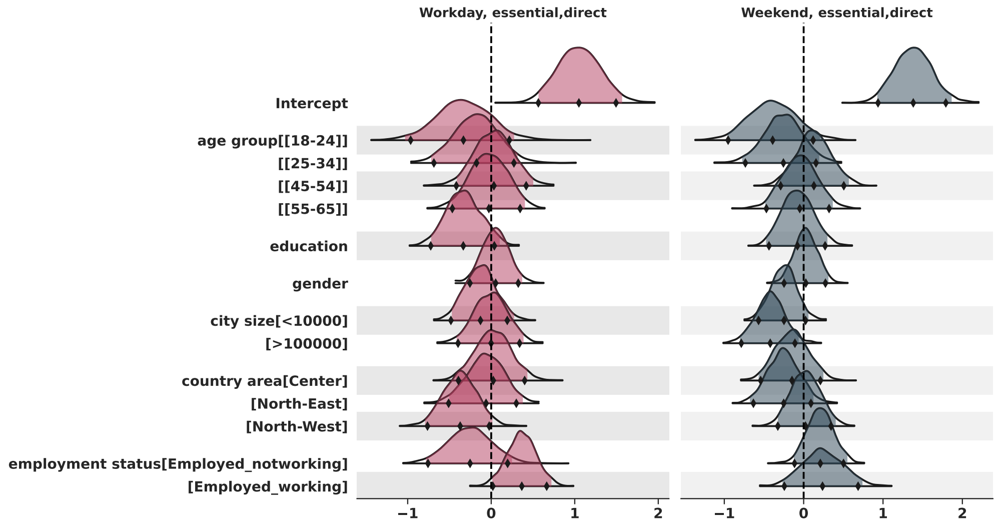

Fig J. Highest Density Interval (HDI) of the posterior distribution for the coefficients (β) from the Bayesian negative binomial model for the number of **direct** contacts during **essential activities**, on weekend and weekday. (Details as in Fig H)

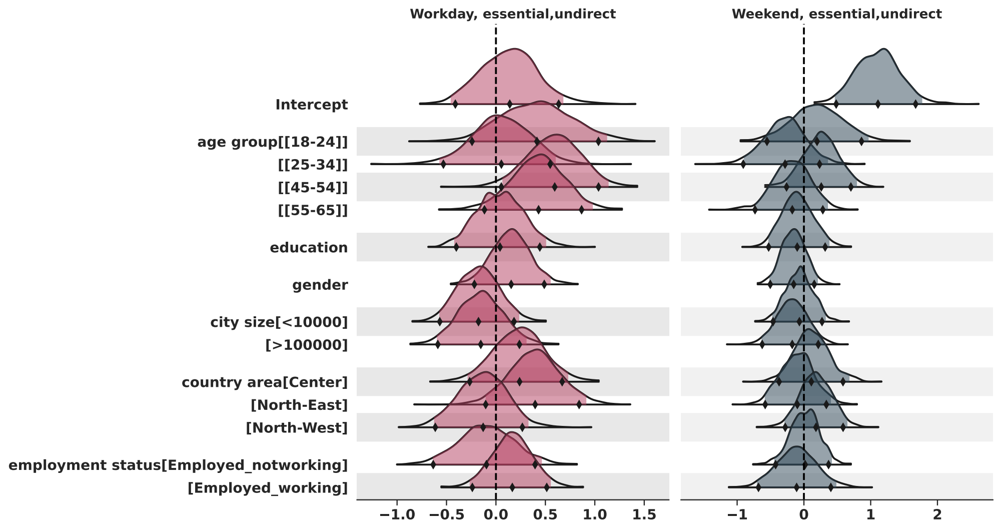

Fig K. Highest Density Interval (HDI) of the posterior distribution for the coefficients (β) from the Bayesian negative binomial model for the number of **indirect** contacts during **essential activities**, on weekend and weekday. (Details as in Fig H)

	Mean	HDI 2.5%	HDI 97.5%
alpha	0.19	0.16	0.21
Intercept	1.05	0.57	1.56
age_group[[18-24]]	-0.33	-1.03	0.27
age_group[[25-34]]	-0.18	-0.72	0.31
age_group[[45-54]]	0.03	-0.41	0.50
age_group[[55-65]]	-0.03	-0.47	0.40
education[Degree]	-0.33	-0.71	0.10
gender[Male]	0.05	-0.26	0.37
city_size[<10000]	-0.13	-0.47	0.27
city_size[>100000]	-0.00	-0.41	0.38
country_area[Center]	0.03	-0.42	0.43
country_area[North-East]	-0.07	-0.50	0.37
country_area[North-West]	-0.37	-0.79	0.01
employed_working[Employed_notworking]	-0.26	-0.78	0.24
employed_working[employed]	0.36	0.02	0.72

Table H. Results of the Negative Binomial Model for direct contacts on weekdays: essential

Weekday direct contacts

	Mean	HDI 2.5%	HDI 97.5%
alpha	0.15	0.13	0.17
Intercept	0.13	-0.45	0.68
age_group[[18-24]]	0.42	-0.24	1.12
age_group[[25-34]]	0.05	-0.57	0.60
age_group[[45-54]]	0.59	0.07	1.14
age_group[[55-65]]	0.42	-0.10	0.98
education[Degree]	0.04	-0.42	0.51
gender[Male]	0.15	-0.22	0.55
city_size[<10000]	-0.18	-0.57	0.23
city_size[>100000]	-0.15	-0.59	0.31
country_area[Center]	0.24	-0.28	0.73
country_area[North-East]	0.40	-0.10	0.91
country_area[North-West]	-0.14	-0.62	0.33
employed_working[Employed_notworking]	-0.10	-0.64	0.46
employed_working[employed]	0.16	-0.26	0.55

Table I. Results of the Negative Binomial Model for indirect contacts on weekdays: essential

Weekday indirect contacts

	Mean	HDI 2.5%	HDI 97.5%
alpha	0.22	0.19	0.24
Intercept	1.38	0.93	1.86
age_group[[18-24]]	-0.39	-0.96	0.20
age_group[[25-34]]	-0.26	-0.77	0.19
age_group[[45-54]]	0.13	-0.30	0.56
age_group[[55-65]]	-0.05	-0.48	0.36
education[Degree]	-0.07	-0.47	0.30
gender[Male]	0.03	-0.25	0.31
city_size[<10000]	-0.25	-0.59	0.06
city_size[>100000]	-0.42	-0.80	-0.07
country_area[Center]	-0.15	-0.56	0.24
country_area[North-East]	-0.25	-0.67	0.12
country_area[North-West]	0.03	-0.32	0.40
employed_working[Employed_notworking]	0.21	-0.12	0.55
employed_working[employed]	0.24	-0.26	0.74

Table J. Results of the Negative Binomial Model for direct contacts on weekends: essential

Weekend direct contacts

	Mean	HDI 2.5%	HDI 97.5%
alpha	0.14	0.12	0.16
Intercept	1.11	0.47	1.77
age_group[[18-24]]	0.20	-0.57	0.96
age_group[[25-34]]	-0.29	-0.90	0.36
age_group[[45-54]]	0.26	-0.25	0.79
age_group[[55-65]]	-0.18	-0.72	0.36
education[Degree]	-0.09	-0.53	0.38
gender[Male]	-0.15	-0.51	0.20
city_size[<10000]	-0.07	-0.47	0.32
city_size[>100000]	-0.18	-0.63	0.28
country_area[Center]	0.12	-0.39	0.68
country_area[North-East]	-0.10	-0.58	0.40
country_area[North-West]	0.18	-0.28	0.64
employed_working[Employed_notworking]	0.01	-0.44	0.41
employed_working[employed]	-0.11	-0.70	0.48

Table K. Results of the Negative Binomial Model for indirect contacts on weekends: essential

Weekend indirect contacts

4.3 Leisure activities

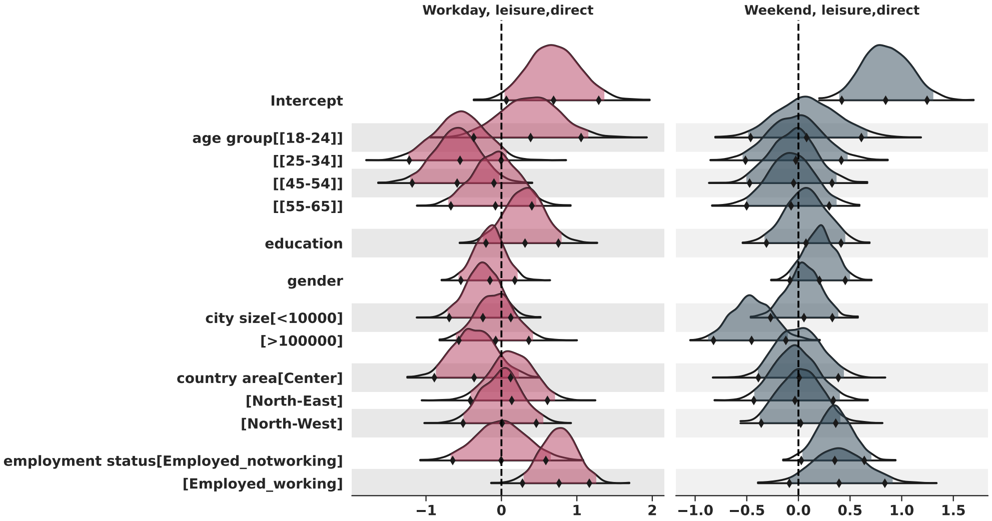

Fig L. Highest Density Interval (HDI) of the posterior distribution for the coefficients (β) from the Bayesian negative binomial model for the number of **direct** contacts during **leisure activities**, on weekend and weekday. (Details as in Fig H)

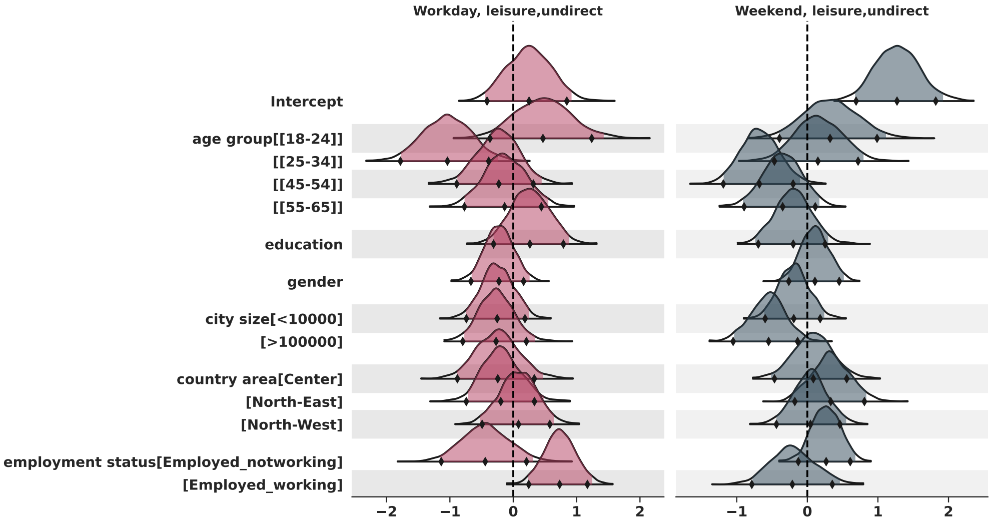

Fig M. Highest Density Interval (HDI) of the posterior distribution for the coefficients (β) from the Bayesian negative binomial model for the number of **indirect** contacts during **leisure activities**, on weekend and weekday. (Details as in Fig H)

	Mean	HDI 2.5%	HDI 97.5%
alpha	0.12	0.10	0.14
Intercept	0.7	0.05	1.36
age_group[[18-24]]	0.38	-0.39	1.15
age_group[[25-34]]	-0.56	-1.23	0.07
age_group[[45-54]]	-0.59	-1.2	-0.03
age_group[[55-65]]	-0.09	-0.7	0.46
education[Degree]	0.31	-0.24	0.79
gender[Male]	-0.16	-0.56	0.21
city_size[<10000]	-0.25	-0.71	0.17
city_size[<100000]	-0.08	-0.6	0.41
country_area[Center]	-0.36	-0.86	0.23
country_area[North-East]	0.14	-0.4	0.70
country_area[North-West]	0.0	-0.5	0.55
employed_working_working[Employed_notworking]	0.0	-0.67	0.65
employed_working_working[Employed_working]	0.76	0.3	1.25

Table L. Results of the Negative Binomial Model for direct contacts on weekdays: leisure activities

Weekday direct contacts

	Mean	HDI 2.5%	HDI 97.5%
alpha	0.10	0.08	0.12
Intercept	0.25	-0.44	0.92
age_group[[18-24]]	0.47	-0.34	1.42
age_group[[25-34]]	-1.04	-1.77	-0.24
age_group[[45-54]]	-0.24	-0.88	0.44
age_group[[55-65]]	-0.13	-0.76	0.55
education[Degree]	0.27	-0.30	0.88
gender[Male]	-0.22	-0.65	0.25
city_size[<10000]	-0.25	-0.74	0.24
city_size[>100000]	-0.27	-0.77	0.34
country_area[Center]	-0.24	-0.88	0.46
country_area[North-East]	-0.19	-0.71	0.47
country_area[North-West]	0.08	-0.52	0.64
employed_working[Employed_notworking]	-0.43	-1.16	0.30
employed_working[employed]	0.74	0.24	1.24

Table M. Results of the Negative Binomial Model for indirect contacts on weekdays: leisure activities

Weekday indirect contacts

	Mean	HDI 2.5%	HDI 97.5%
alpha	0.20	0.18	0.23
Intercept	0.85	0.40	1.30
age_group[[18-24]]	0.09	-0.48	0.67
age_group[[25-34]]	-0.03	-0.54	0.48
age_group[[45-54]]	-0.05	-0.50	0.37
age_group[[55-65]]	-0.07	-0.50	0.37
education[Degree]	0.07	-0.32	0.45
gender[Male]	0.20	-0.08	0.50
city_size[<10000]	0.05	-0.25	0.38
city_size[>100000]	-0.45	-0.87	-0.11
country_area[Center]	0.01	-0.39	0.44
country_area[North-East]	-0.03	-0.42	0.40
country_area[North-West]	0.02	-0.37	0.40
employed_working[Employed_notworking]	0.35	0.04	0.70
employed_working[Employed_working]	0.40	-0.09	0.91

Table N. Results of the Negative Binomial Model for direct contacts on weekends: leisure activities

Weekend direct contacts

	Mean	HDI 2.5%	HDI 97.5%
alpha	0.13	0.11	0.14
Intercept	1.27	0.68	1.92
age_group[[18-24]]	0.33	-0.39	1.11
age_group[[25-34]]	0.16	-0.48	0.79
age_group[[45-54]]	-0.67	-1.18	-0.11
age_group[[55-65]]	-0.35	-0.91	0.17
education[Degree]	-0.20	-0.73	0.29
gender[Male]	0.11	-0.26	0.51
city_size[<10000]	-0.19	-0.60	0.24
city_size[>100000]	-0.56	-1.03	-0.06
country_area[Center]	0.08	-0.51	0.62
country_area[North-East]	0.34	-0.23	0.83
country_area[North-West]	0.04	-0.43	0.55
employed_working[Employed_notworking]	0.26	-0.12	0.68
employed_working[employed]	-0.20	-0.77	0.46

Table O. Results of the Negative Binomial Model for indirect contacts on weekend: leisure activities

Weekend indirect contacts

4.3.1 Transport

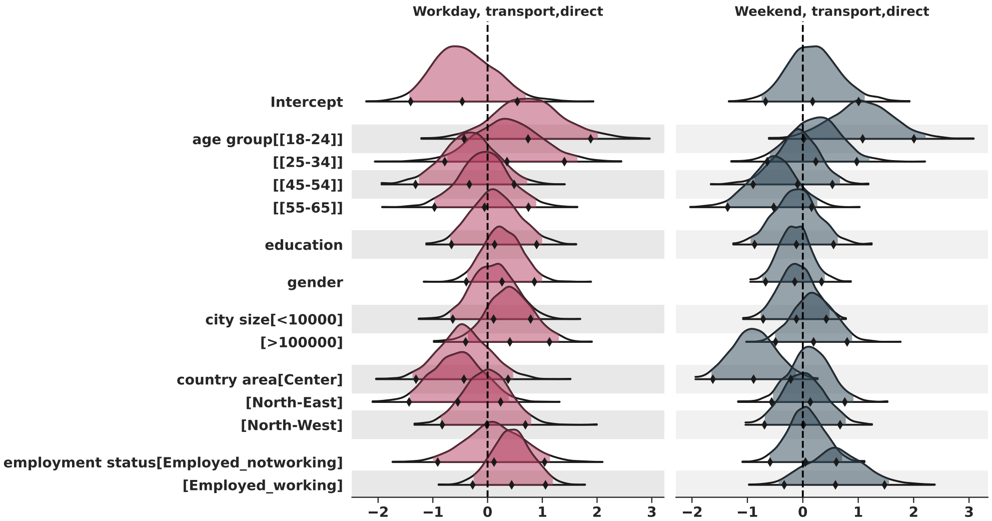

Fig N. Highest Density Interval (HDI) of the posterior distribution for the coefficients (β) from the Bayesian negative binomial model for the number of **direct** contacts during **transport**, on weekend and weekday. (Details as in Fig H)

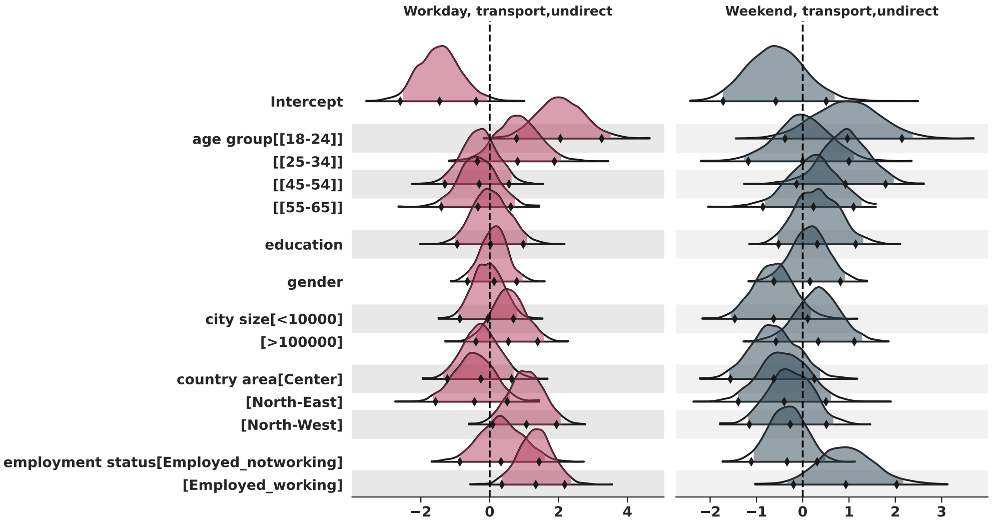

Fig O. Highest Density Interval (HDI) of the posterior distribution for the coefficients (β) from the Bayesian negative binomial model for the number of **indirect** contacts during **transport**, on weekend and weekday. (Details as in Fig H)

	Mean	HDI 2.5%	HDI 97.5%
alpha	0.05	0.04	0.06
Intercept	-0.42	-1.42	0.69
age_group[[18-24]]	0.76	-0.54	2.01
age_group[[25-34]]	0.37	-0.74	1.64
age_group[[45-54]]	-0.34	-1.25	0.72
age_group[[55-65]]	-0.05	-0.97	0.89
education[Degree]	0.14	-0.68	0.99
gender[Male]	0.27	-0.37	0.99
city_size[<10000]	0.11	-0.68	0.85
city_size[>100000]	0.41	-0.35	1.30
country_area[Center]	-0.42	-1.37	0.46
country_area[North-East]	-0.55	-1.42	0.38
country_area[North-West]	-0.02	-0.85	0.79
employed_working[Employed_notworking]	0.12	-0.99	1.14
employed_working[employed]	0.44	-0.25	1.19

Table P. Results of the Negative Binomial Model for direct contacts on weekdays: transport

Weekday direct contacts

	Mean	HDI 2.5%	HDI 97.5%
alpha	0.04	0.03	0.05
Intercept	-1.45	-2.51	-0.08
age_group[[18-24]]	2.06	0.82	3.50
age_group[[25-34]]	0.82	-0.37	2.06
age_group[[45-54]]	-0.31	-1.36	0.63
age_group[[55-65]]	-0.33	-1.44	0.73
education[Degree]	0.04	-0.99	1.08
gender[Male]	0.12	-0.63	0.95
city_size[<10000]	-0.05	-0.90	0.78
city_size[>100000]	0.54	-0.38	1.58
country_area[Center]	-0.25	-1.34	0.69
country_area[North-East]	-0.45	-1.65	0.59
country_area[North-West]	1.07	0.06	2.05
employed_working[Employed_notworking]	0.34	-0.86	1.59
employed_working[employed]	1.33	0.36	2.35

Table Q. Results of the Negative Binomial Model for indirect contacts on weekdays: transport

Weekday indirect contacts

	Mean	HDI 2.5%	HDI 97.5%
alpha	0.07	0.05	0.08
Intercept	0.19	-0.74	1.11
age_group[[18-24]]	1.08	0.03	2.21
age_group[[25-34]]	0.22	-0.59	1.19
age_group[[45-54]]	-0.11	-0.86	0.67
age_group[[55-65]]	-0.54	-1.37	0.26
education[Degree]	-0.12	-0.93	0.62
gender[Male]	-0.14	-0.72	0.39
city_size[<10000]	-0.11	-0.76	0.48
city_size[>100000]	0.20	-0.50	0.88
country_area[Center]	-0.88	-1.64	-0.13
country_area[North-East]	0.14	-0.53	0.91
country_area[North-West]	0.02	-0.68	0.77
employed_working[Employed_notworking]	0.05	-0.59	0.70
employed_working[employed]	0.60	-0.43	1.55

Table R. Results of the Negative Binomial Model for direct contacts on weekends: transport

Weekend direct contacts

	Mean	HDI 2.5%	HDI 97.5%
alpha	0.05	0.04	0.06
Intercept	-0.57	-1.72	0.68
age_group[[18-24]]	0.96	-0.36	2.37
age_group[[25-34]]	-0.00	-1.27	1.10
age_group[[45-54]]	0.92	-0.11	1.96
age_group[[55-65]]	0.22	-0.82	1.26
education[Degree]	0.33	-0.54	1.30
gender[Male]	0.15	-0.66	0.91
city_size[<10000]	-0.63	-1.55	0.17
city_size[>100000]	0.33	-0.56	1.28
country_area[Center]	-0.61	-1.58	0.37
country_area[North-East]	-0.39	-1.40	0.61
country_area[North-West]	-0.27	-1.17	0.66
employed_working[Employed_notworking]	-0.34	-1.05	0.48
employed_working[employed]	0.95	-0.32	2.16

Table S. Results of the Negative Binomial Model for indirect contacts on weekend: transport

Weekend indirect contacts

4.4 Health activities

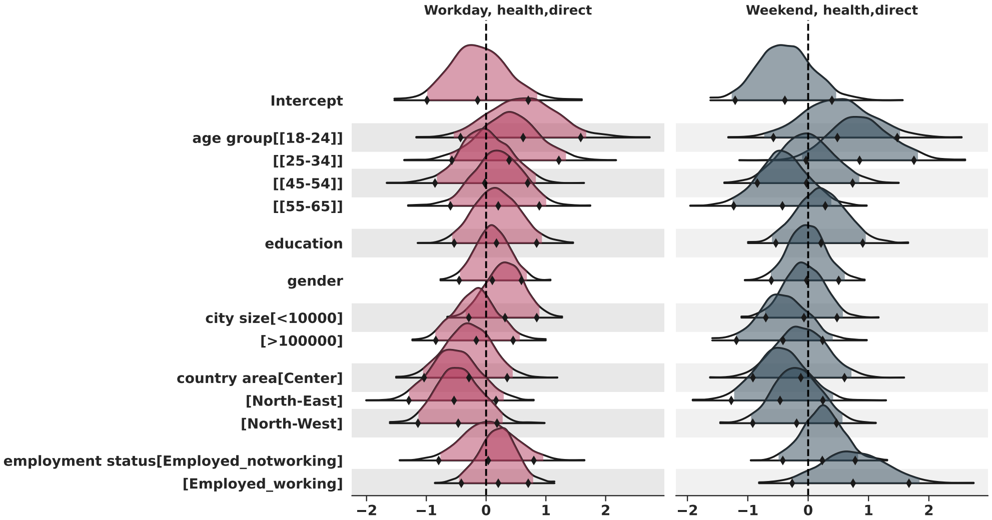

Fig P. Highest Density Interval (HDI) of the posterior distribution for the coefficients (β) from the Bayesian negative binomial model for the number of **direct** contacts in **health locations**, on weekend and weekday. (Details as in Fig H)

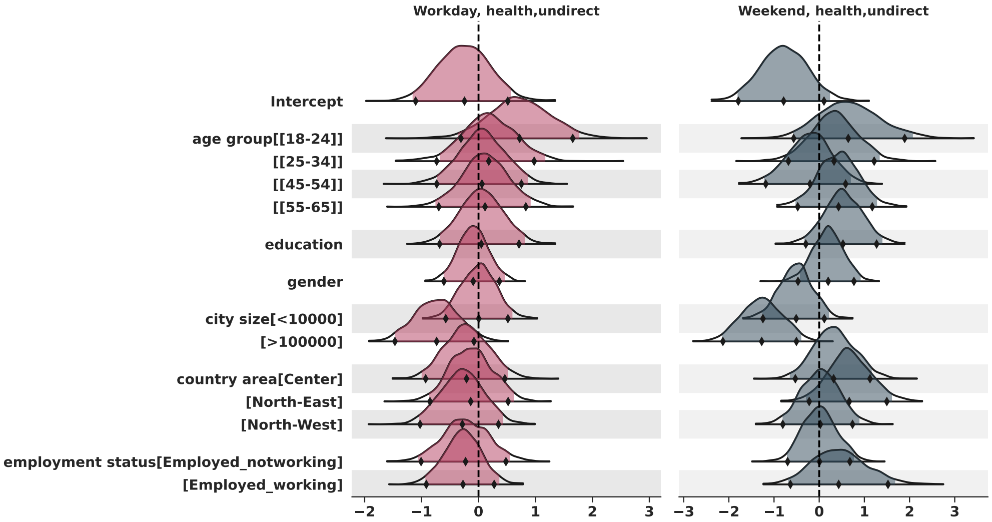

Fig Q. Highest Density Interval (HDI) of the posterior distribution for the coefficients (β) from the Bayesian negative binomial model for the number of **indirect** contacts in **health locations**, on weekend and weekday. (Details as in Fig H)

	Mean	HDI 2.5%	HDI 97.5%
alpha	0.06	0.05	0.08
Intercept	-0.12	-0.98	0.85
age_group[[18-24]]	0.62	-0.54	1.67
age_group[[25-34]]	0.38	-0.60	1.33
age_group[[45-54]]	-0.02	-0.82	0.83
age_group[[55-65]]	0.20	-0.62	1.00
education[Degree]	0.18	-0.56	0.93
gender[Male]	0.10	-0.43	0.68
city_size[<10000]	0.32	-0.34	0.89
city_size[>100000]	-0.17	-0.84	0.56
country_area[Center]	-0.29	-1.05	0.44
country_area[North-East]	-0.53	-1.28	0.29
country_area[North-West]	-0.46	-1.13	0.27
employed_working[Employed_notworking]	0.04	-0.76	0.95
employed_working[employed]	0.20	-0.45	0.78

Table T. Results of the Negative Binomial Model for direct contacts on weekdays: health

Weekday direct contacts

	Mean	HDI 2.5%	HDI 97.5%
alpha	0.07	0.06	0.09
Intercept	-0.25	-1.16	0.57
age_group[[18-24]]	0.73	-0.36	1.77
age_group[[25-34]]	0.18	-0.67	1.17
age_group[[45-54]]	0.06	-0.73	0.86
age_group[[55-65]]	0.11	-0.75	0.91
education[Degree]	0.06	-0.67	0.81
gender[Male]	-0.09	-0.60	0.46
city_size[<10000]	0.00	-0.60	0.59
city_size[>100000]	-0.73	-1.46	0.01
country_area[Center]	-0.20	-0.99	0.51
country_area[North-East]	-0.13	-0.86	0.62
country_area[North-West]	-0.29	-1.04	0.43
employed_working[Employed_notworking]	-0.22	-1.06	0.55
employed_working[employed]	-0.28	-0.93	0.36

Table U. Results of the Negative Binomial Model for indirect contacts on weekdays: health

Weekday indirect contacts

	Mean	HDI 2.5%	HDI 97.5%
alpha	0.06	0.05	0.07
Intercept	-0.38	-1.26	0.46
age_group[[18-24]]	0.49	-0.72	1.52
age_group[[25-34]]	0.87	-0.10	1.82
age_group[[45-54]]	-0.02	-0.88	0.84
age_group[[55-65]]	-0.42	-1.25	0.38
education[Degree]	0.22	-0.60	0.95
gender[Male]	-0.02	-0.61	0.60
city_size[<10000]	-0.07	-0.71	0.57
city_size[>100000]	-0.41	-1.18	0.40
country_area[Center]	-0.12	-0.93	0.71
country_area[North-East]	-0.46	-1.22	0.41
country_area[North-West]	-0.19	-0.94	0.56
employed_working[Employed_notworking]	0.23	-0.48	0.82
employed_working[employed]	0.76	-0.26	1.84

Table V. Results of the Negative Binomial Model for direct contacts on weekends: health

Weekend direct contacts

	Mean	HDI 2.5%	HDI 97.5%
alpha	0.06	0.04	0.07
Intercept	-0.78	-1.80	0.23
age_group[[18-24]]	0.67	-0.57	2.07
age_group[[25-34]]	0.33	-0.72	1.33
age_group[[45-54]]	-0.22	-1.21	0.69
age_group[[55-65]]	0.42	-0.52	1.27
education[Degree]	0.54	-0.32	1.39
gender[Male]	0.19	-0.42	0.91
city_size[<10000]	-0.52	-1.25	0.21
city_size[>100000]	-1.27	-2.14	-0.40
country_area[Center]	0.33	-0.64	1.16
country_area[North-East]	0.68	-0.29	1.60
country_area[North-West]	0.02	-0.80	0.88
employed_working[Employed_notworking]	0.01	-0.71	0.76
employed_working[Employed]	0.46	-0.68	1.68

Table W. Results of the Negative Binomial Model for indirect contacts on weekend: health

Weekend indirect contacts

References Cited in Supplementary Information

References

1. Capretto T, Piho C, Kumar R, Westfall J, Yarkoni T, Martin OA. Bambi: A Simple Interface for Fitting Bayesian Linear Models in Python. Journal of Statistical Software. 2022;103:1–29. doi:10.18637/jss.v103.i15.
2. van de Schoot R, Depaoli S, King R, Kramer B, Märtens K, Tadesse MG, et al. Bayesian statistics and modelling. Nature Reviews Methods Primers. 2021;1(1):1. doi:10.1038/s43586-020-00001-2.
3. Coletti P, Wambua J, Gimma A, Willem L, Vercruysse S, Vanhoutte B, et al. CoMix: comparing mixing patterns in the Belgian population during and after lockdown. Scientific reports. 2020;10(1):1–10.
4. Jarvis CI, Van Zandvoort K, Gimma A, Prem K, Klepac P, Rubin GJ, et al. Quantifying the impact of physical distance measures on the transmission of COVID-19 in the UK. BMC medicine. 2020;18(1):1–10.
5. Sammut C. Markov Chain Monte Carlo. In: Sammut C, Webb GI, editors. Encyclopedia of Machine Learning. Boston, MA: Springer US; 2010. p. 639–642. Available from: https://doi.org/10.1007/978-0-387-30164-8_511.
