# Supplementary material for: Socioeconomic determinants of protective behaviors and contact patterns in the post-COVID-19 pandemic era: A cross-sectional study in Italy
Source: PLoS Comput Biol. 2025 Aug 4;21(8):e1013262. doi: 10.1371/journal.pcbi.1013262 (PMC12338834; doi:10.1371/journal.pcbi.1013262)
Supplement: S2 Text — (PDF) [file pcbi.1013262.s002.pdf]

Appendix: Survey Instrument Details

This appendix provides a detailed description of the questions included in the survey instrument, grouped by topic.

Sociodemographics

A1 Gender: Participant's self-reported gender. *Options:* Male; Female.

A2 Age: Participant's age in years. *Options:* Numerical input. (Note: Grouped for analysis).

A3 Municipality Size: Size category of the participant's municipality of residence, derived from ISTAT database based on specific municipality provided by participant. *Options (recoded):* Less than 10,000 inhabitants; Between 10,000 and 100,000 inhabitants; Over 100,000 inhabitants.

A5 Education Level: Highest educational qualification obtained by the participant. *Options:* Post-graduate degree (PhD/Master's); University degree (5-year program or equivalent); University degree (3-year program); High school diploma; Middle school certificate; Elementary school certificate; No qualification. (Note: Recoded to Degree/No Degree for analysis).

A6 Employment Status: Participant's current primary occupation/employment status. *Options:* Employed; Unemployed. (Note: Used for analysis and for conditional display of subsequent questions).

Vaccination and Protective Behaviours

D1 Influenza Vaccine: Question: "In the last 6 months, have you had the flu vaccine?" *Options:* Yes; No; Prefer not to answer.

D2 COVID-19 Vaccine: Question: "Have you had the Covid-19 vaccine? If so, how many doses have you had?" *Options:* 1; 2; 3; More than 3; No, none; Prefer not to answer. (Note: Mapped to Yes/No for analysis).

D3 NPI Use (Last Month): Question: "In the last month, have you used the following preventive measures against infectious diseases (Covid-19 and/or flu)?" Response scale applied to both items below. *Scale:* Always; Sometimes; Never.

- Protective mask
- Social distancing

Perceived Impact of COVID-19

D4 Perceived Impact: Participants read statements about the impact of Covid-19 and indicated agreement based on their personal situation. *Scale:* 5-point Likert scale (1 = "not at all agree" to 5 = "strongly agree"). Applied to the following four statements:

- "Covid has had a negative impact on my economic situation"
- "Covid has had a negative impact on my social situation (e.g. I've lost contact with friends, I participate less in social events, etc.)"
- "Covid has had a negative impact on my psychological condition (e.g. I feel more mentally fatigued, I feel more stressed...)"
- "Covid has had a negative impact on my work situation (e.g. I lost my job, it was more difficult to find employment)"

Contact Diary Setup Questions

D8 Work Status (Sunday): Question: "Did you work last Sunday?" *Options:* Yes; No. (Note: Conditions the display of work/study location in D9/D10).

D11 Work Status (Weekday): Question: "Did you work last weekday (Monday)?" *Options:* Yes; No. (Note: Conditions the display of work/study location in D12/D13).

Contact Diary Details (Sunday and Monday)

Participants were asked to report contacts for the previous Sunday and Monday (reference day: Tuesday).

Direct Contacts (Questions D9 & D12)

Definition: People met in person with whom the participant exchanged at least a few words OR had physical contact (e.g., handshake, hug, kiss, contact sport). Excluded interactions only by phone or online. *Task:* For each specified location, indicate how many people in specific age groups were contacted directly. *Response Scale:* Numerical count from 0 to 100 for each age group within each location. *Age Groups of Contacts:* 0-5, 6-17, 18-24, 25-35, 36-45, 46-55, 56-65, 65+. *Locations Assessed:*

- Inside own home (excluding members of own family)
- In workplaces or study (work/school/university) [Conditional on D8/D11=Yes or A6=Student]
- On public transport
- Performing essential activities (e.g., grocery shopping, etc.)

- Performing health-related activities (medical visits, hospitals, laboratory tests)
- Performing leisure activities (sports, concerts, events, restaurants, etc.)

Indirect Contacts (Questions D10 & D13 - Implied)

Definition: People with whom the participant spent more than 15 minutes in the same place (e.g., on public transport) AND maintained a distance of 1.5 meters or less, WITHOUT direct interaction (words/touch). Excluded direct contacts already reported and interactions only by phone or online. *Task:* For each specified location, indicate how many people were contacted indirectly. *Response Scale:* Numerical count from 0 to 100 for each location. *Locations Assessed:*

- Inside own home (excluding members of own family)
- In workplaces or study (work/school/university) [Conditional on D8/D11=Yes or A6=Student]
- On public transport
- Performing essential activities (e.g., grocery shopping, etc.)
- Performing health-related activities (medical visits, hospitals, laboratory tests)
- Performing leisure activities (sports, concerts, events, restaurants, etc.)
